# Plasma Functionalization Enables Diffusion Control of Reactive Oxygen Species

**DOI:** 10.1002/smll.202502311

**Published:** 2025-07-11

**Authors:** Paula Navascués, Flaela Kalemi, Flavia Zuber, Philipp Meier, Ludovica M. Epasto, Michał Góra, Barbara Hanselmann, Svetlana Kucher, Enrica Bordignon, Qun Ren, Giacomo Reina, Dirk Hegemann

**Affiliations:** ^1^ Laboratory for Advanced Fibers Empa Swiss Federal Laboratories for Materials Science and Technology Lerchenfeldstrasse 5 St. Gallen 9014 Switzerland; ^2^ Laboratory for Biointerfaces Empa Swiss Federal Laboratories for Materials Science and Technology Lerchenfeldstrasse 5 St. Gallen 9014 Switzerland; ^3^ Laboratory for Nanomaterials in Health Empa Swiss Federal Laboratories for Materials Science and Technology Lerchenfeldstrasse 5 St. Gallen 9014 Switzerland; ^4^ Department of Physical Chemistry University of Geneva 30 Quai Ernest Ansermet Genèva 1211 Switzerland; ^5^ Department of Materials ETH Zürich Zürich 8093 Switzerland

**Keywords:** chemodynamics, plasma technology, reactive oxygen species (ROS), singlet oxygen, superoxide

## Abstract

Reactive oxygen species (ROS) are promising green candidates for tackling challenges ranging from antimicrobial resistance to water decontamination. Metal oxide nanomaterials structured as thin films, deposited at room temperature (RT) using plasma technology, can deliver ROS to the environment by catalyzing oxygen and water following a chemodynamics approach. This study proposes thin film plasma polymerization as a strategy to precisely control ROS delivery, unravel ROS formation mechanism at the catalytic interface, and ensure ROS‐driven chemistry. A proper combination of semiconductors, specifically silver oxide and titanium oxide, is used as a model system for ROS production. This specific coupling of semiconductors produces ROS in the dark due to charge separation without ion leaching. Plasma surface functionalization with nanoporous SiOx‐like films in the 1–100 nm range allows selective control of the delivery of radicals with different characteristic lifetimes such as superoxide anion and singlet oxygen based on the thickness of the functional layer. As proof of promising applications, results regarding radicals' detection are correlated with the antimicrobial activity of the ROS‐releasing system. Thin film plasma surface functionalization allows control of ROS delivery, ensuring that the material efficacy is due to ROS and not by other direct redox chemistry or leaching processes.

## Graphical abstract

Plasma surface functionalization of thin films enables controlled delivery of reactive oxygen species (ROS) generated by catalytic coatings. This approach ensures efficacy driven by radical activity rather than direct redox reactions or leaching. The selective release of radicals with distinct lifetimes is linked to both antimicrobial and antiviral effects.

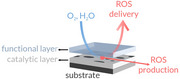

## 1 Introduction

Biocompatible nanocatalysts have emerged over the last decade as a key tool for addressing a variety of global problems. In this context, oxidases mimicking nanostructures have been studied for their catalytic capacity to convert O_2_ into reactive oxygen species (ROS).^[^ ^1^ ^]^ ROS include not only free radicals, such as singlet oxygen (^1^O_2_), hydroxyl radical (•OH), and superoxide radical (O_2_^•–^), but also non‐radical species such as hydrogen peroxide (H_2_O_2_).^[^ ^2^ ^]^ These highly reactive, oxidizing species enable a range of applications from antimicrobial^[^ ^3^ ^]^ and cancer treatment^[^ ^4^ ^]^ to the degradation of organic contaminants such as pollutant removal^[^ ^5^ ^]^ or water decontamination.^[^ ^6^ ^]^ The simplicity of ROS compared to complex chemicals such as drugs and other oxidizing substances makes them specific, clean, and green candidates for numerous applications, as they avoid, e.g., chlorine or other dangerous side products. In terms of antibacterial and antiviral application, ROS‐generating nanomaterials serve as broad‐range disinfectants, genuinely beyond antibiotics, making them also ideal candidates for combating antimicrobial resistance (AMR).^[^ ^3^ ^]^ In this regard, their activity is associated with oxidative stress, damaging cellular components such as lipids, proteins, and DNA, leading to cell death or, at least, growth inhibition.^[^ ^7^ ^]^ There are plenty of materials capable of producing ROS, mainly due to the catalytic properties of nanomaterials of different dimensions^[^ ^8^ ^]^ but also strategies such as hydrogels^[^ ^9^ ^]^ and living‐materials.^[^ ^10^ ^]^ Common strategies in biomedicine, focusing on therapy, are based on ROS production by nanoparticles (i.e., generally 0D materials).^[^ ^11^, ^12^ ^]^ In this context, ROS are mainly generated by released metal ions (e.g., Ag^+^) that interfere with the bacterial metabolism. There are, however, fewer studies reporting ROS‐producing thin film surfaces for therapeutic approaches, despite such systems have been deeply studied by the photocatalysis community in terms of energy and environmental applications.^[^ ^13^, ^14^ ^]^ Thus, exploring new approaches by connecting nano‐catalysts, biology, and medicine is becoming more and more attractive.

Considering the promising role of ROS for targeted applications, advanced materials capable of producing and precisely controlling the delivery of these active species are desired. ROS can be produced when catalytic active surfaces come into contact with oxygen, and even water molecules.^[^ ^8^ ^]^ ROS are produced at the catalyst surface and immediately delivered to the environment, rapidly reacting with any substance that can be oxidized. Specific control of the type of ROS produced can be tailored, e.g., by applying stimuli such as light^[^ ^15^ ^]^ or heat.^[^ ^16^ ^]^ During the last few years, remarkable progress has been made toward the specific production of ROS such as singlet oxygen (^1^O_2_) via light activation of graphene oxide^[^ ^17^ ^]^ or •OH radicals via Fenton‐like reactions (chemodynamic therapy),^[^ ^18^ ^]^ among others. The controlled diffusion of these radicals into the target media once they are produced, however, has been poorly studied.

Functionalizing ROS‐producing surfaces offers a promising strategy for controlling ROS delivery. In this regard, state‐of‐the‐art results generally consist of the incorporation of functional coatings onto TiO_2_‐based nanoparticles via wet chemical methods,^[^ ^5^, ^19^, ^20^, ^21^, ^22^ ^]^ although reverse approaches have been also considered, e.g., incorporating TiO_2_ on silica nanoparticles^[^ ^23^ ^]^ or directly on polymeric substrates.^[^ ^24^ ^]^ K.J. Heo et al. reported the functionalization of doped TiO_2_ nanoparticles with hydrophobic PFOTES via an aerosol technique to ensure the stability of the system against moisture.^[^ ^19^ ^]^ Polydimethylsiloxane (PDMS) is the most commonly reported functional polymer used to ensure the mechanical stability and durability of nanoparticles.^[^ ^5^, ^20^ ^]^ R. Ghosh et al. recently reported the combination of hydrophilic and hydrophobic functionalization of TiO_2_ nanoparticles to demonstrate wettability control for water harvesting.^[^ ^6^ ^]^ Regarding the functionalization of thin film structures, Liu et al. published the incorporation of PDMS grafting onto TiO_2_ thin films.^[^ ^21^ ^]^ The authors varied the grafting thickness between 0.6 and 5.5 nm and activated the material with UV light. They found a decreasing tendency for the degradation of a generic dye with respect to the grafting thickness, claiming that at 5.5 nm the photocatalytic activity was blocked by the PDMS layer. All these studies demonstrate that catalytic materials can retain at least a degree of activity when subjected to surface functionalization. However, it is not clearly discussed how the activity is tailored and what the mechanisms behind it are. Most of the functional coatings are intrinsically hydrophobic, thereby limiting or delaying the activity of the materials when H_2_O is necessary for ROS production. Additionally, due to the high phototoxicity and low tissue permeation depth, UV light should be avoided for clinical applications. Likewise, ROS production requiring UV lamps for water purification has been associated with high energy consumption.^[^ ^25^ ^]^ These facts justify the increasing interest in nanomaterials producing ROS by visible and IR light excitation.^[^ ^16^, ^17^ ^]^ Hence, the preparation of a catalytic, non‐metal‐releasing thin film with a controlled wide activity is highly desirable for many applications ranging from medicine to sustainability.

Defective TiO_2_‐type thin films are established as a robust solution for chemodynamic applications.^[^ ^26^ ^]^ Based on that, we reported AgOx‐doped TiOx thin films (i.e., AgOx/TiOx) fabricated by low‐pressure plasma technology as a rapid antibacterial solution based on ROS generation.^[^ ^27^ ^]^ The efficacy of this material was tested against gram‐positive and gram‐negative bacteria, and its activity upon storage in the dark and after daylight reactivation was followed over two years, showing a robust and reproducible behavior avoiding any side effects for human cells.^[^ ^27^ ^]^ In antimicrobial applications, maintaining ROS homeostasis is crucial: an excessive amount of radicals can activate inflammatory pathways in healthy tissues, while low catalytic efficiency may hinder the efficacy.^[^ ^7^ ^]^ The present study uses the combination of silver oxide (AgOx) and titanium oxide (TiOx) semiconductors as a model thin film system for ROS generation. Plasma functionalization using plasma polymer films (PPFs) (up to 100 nm thick) deposited on top of the catalytic surface has been investigated as a strategy to precisely control ROS delivery, unravel the ROS formation mechanism at the catalytic interface, and ensure ROS‐driven chemistry. An in‐depth characterization of the mechanisms behind the production and delivery of ROS is performed by using fluorescence spectroscopy and electron paramagnetic resonance (EPR). As proof of promising applications, the detection of radicals has been associated with the antimicrobial activity of the system. To the best of our knowledge, this study is the first to report such a precise quantitative and qualitative controlled release of ROS produced at the surface of catalytic thin films.

## 2 Results and Discussion

### 2.1 Fabrication and Functionalization of Catalytic Plasma Coatings

A previous study by our research group thoroughly investigated on the role of catalytic plasma coatings consisting of AgOx‐doped amorphous TiOx, i.e., AgOx/TiOx, as antibacterial agents based on ROS generation.^[^ ^27^ ^]^ The plasma sputtering process yields a nonstoichiometric oxidation state of TiOx, stabilized by oxygen vacancies (O_V_), while the plasma post‐oxidation step affixes the AgOx structures, avoiding the release of Ag ions.^[^ ^28^ ^]^ The AgOx‐doping incorporates silver oxide nanoislets, which act as catalytic active sites and modify the band gap of the material.^[^ ^29^ ^]^ The activity of the catalytic plasma coating was demonstrated after daylight activation, without the need for specific UV activation or high‐intensity lamps. A clear correlation was found between the production of ROS – generated when the surface is in contact with water and oxygen – and the material's antibacterial properties, as demonstrated by testing its activity after storage in the dark for up to two years. Building on these previous findings, the current study selects an optimized AgOx/TiOx system as the ROS‐producing platform.

**Scheme 1:**
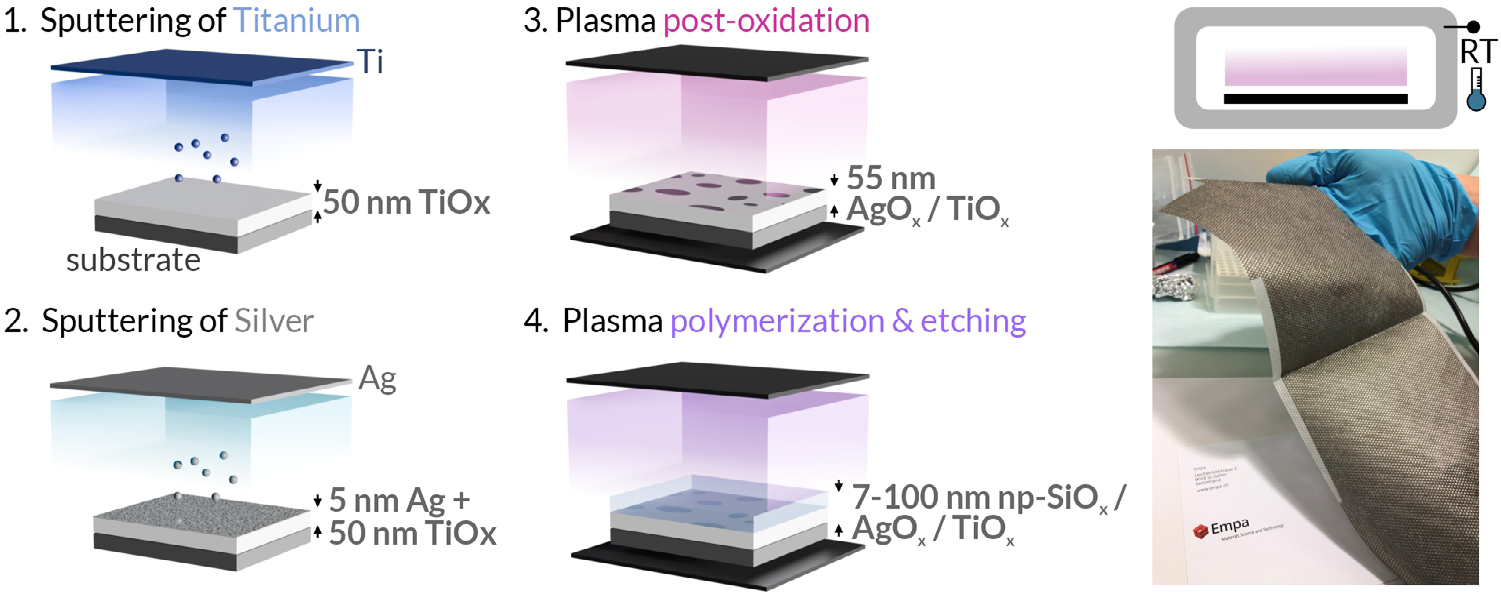
Fabrication of catalytic coatings and its functionalization at RT by a multistep plasma process (steps 1 to 4): 1) sputtering from a titanium target to fabricate the TiOx layer, 50 nm‐thick, 2) sputtering from a silver target to deposit 5 nm of Ag onto the TiOx layer, 3) Ar/O_2_ plasma post‐oxidation to obtain the AgOx/TiOx system, 4) plasma polymerization and etching of HMDSO to functionalize AgOx/TiOx. On the right side, a photograph of a wound dressing coated with the AgOx/TiOx material is shown.

**Scheme** **1** illustrates the multistep plasma process followed to fabricate the catalytic metal oxides (AgOx/TiOx) and its functionalization with nanoporous PPFs (np‐SiOx/AgOx/TiOx). The samples consist of nanoscale thin films produced entirely using low‐pressure plasma technology at RT. Specifically, it consists of a 50 nm‐thick TiOx layer with nominally 5 nm of Ag deposited on top, subsequently sputtering from titanium and silver targets, respectively (Scheme 1, steps 1 and 2). A variety of materials are utilized as substrates, including glass cover slides, silicon wafers, and various textiles/nonwovens such as those used for wound dressings. The operation at RT enables the coating of sensitive substrates without inducing any damage, as can be observed in the photograph of the wound dressing on the right side of Scheme 1 (the gray color corresponds to the AgOx/TiOx coating deposited on the textile). This fact is particularly relevant, taking into account the established application of ROS delivery for wound healing.^[^ ^7^ ^]^ After plasma post‐oxidation (step 3), the AgOx/TiOx catalyst is provided with an average thickness of 55 ± 5 nm. The material has been further functionalized with nanoporous PPFs (here np‐SiOx) of different thickness to control ROS delivery (step 4). This functionalization was carried out by plasma polymerization and etching using hexamethyldisiloxane (HMDSO) and oxygen.^[^ ^30^ ^]^ Thus, nanoporous PPFs with SiOx chemistry^[^ ^30^ ^]^ have been deposited as functional layers varying the thickness between 7 and 100 nm. The PPF shows a superhydrophilic wetting behavior that, together with the nanoporosity, allows the diffusion of water and oxygen molecules through the np‐SiOx layer,^[^ ^30^ ^]^ reaching the plasma polymer‐metal oxide interface where ROS are generated. Afterward, the produced ROS can diffuse through the PPF to the outside, with the functional layer acting as a ROS‐delivery modulator. The plasma polymer structure is resistant to highly oxidizing conditions, as those presented in the ROS environment, because of the stability of the plasma polymer matrix. More details about the fabrication procedure be found in the Experimental Section.

**Figure 1:**
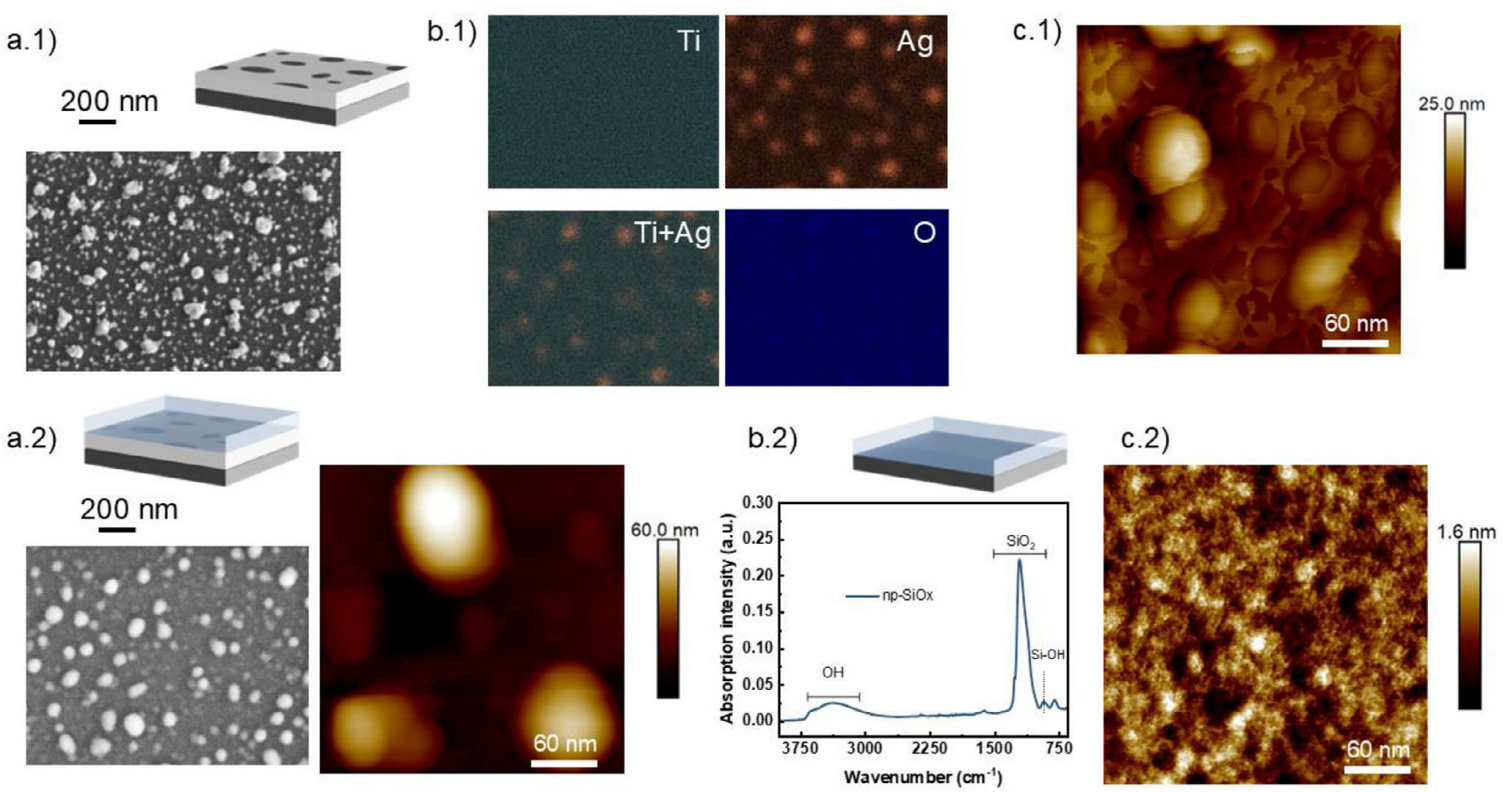
Characterization of the ROS‐producing system (i.e., the catalytic AgOx/TiOx plasma coating) and its functionalization with nanoporous PPFs. a.1) SEM top view micrograph of AgOx/TiOx. b.1) EDS analysis of AgOx/TiOx, including composition maps of titanium (Ti), silver (Ag), Ti and Ag overlapped (Ti+Ag), and oxygen (O). c.1) AFM micrograph of AgOx/TiOx. a.2) np‐SiOx/AgOx/TiOx surface analyzed in top view by (left) SEM and (right) AFM. b.2) ATR‐FTIR characterization of np‐SiOx thin film and c.2) AFM high resolution analysis of its smooth surface.

**Figure** **1** presents the characterization of the AgOx/TiOx system, its functionalization (np‐SiOx/AgOx/TiOx), and just the np‐SiOx functional layer. Silver oxide nanoislets of different sizes (10–100 nm) can be observed dispersed on the TiOx layer (see Figure 1a.1). As mentioned above, these nanointerfaces contribute to the ROS production, acting as catalytic active sites alongside the intrinsic defects.^[^ ^27^ ^]^ Comparable characterization of the TiOx support alone and the system before plasma post‐oxidation (Ag/TiOx) can be found in our previous study, including surface chemistry characterization revealing the presence of oxygen vacancies on the highly nonstoichiometric TiOx layer.^[^ ^27^ ^]^ EDS characterization in Figure 1b.1 clearly reveals the homogeneous and flat TiOx support (Ti), the distribution of the silver oxide nanoislets on it (Ag), the superposition of both elements (Ti+Ag), and the overall presence of oxygen at the surface (O). Atomic force microscope (AFM) analysis in Figure 1c.1 allows to distinguish between the roughness of the TiOx support and the AgOx islets on top, revealing an average roughness of 3.4 nm. Figure 1a.2 presents scanning electron microscopy (SEM) (left) and AFM (right) analysis of the np‐SiOx/AgOx/TiOx system. The results show that the addition of a thin layer of np‐SiOx largely preserves the morphologies previously characterized for the AgOx/TiOx material. The deposition of np‐SiOx slightly affects the Ag nanostructures, inducing additional oxidation, with an average roughness of 9.9 nm as determined by AFM. The micrographs shown in Figure 1a.2 correspond to a np‐SiOx thickness of 15 nm, but similar features were obtained for coatings examined in the 7–100 nm range. The functional layer is conformal, with no cracks or defects, ensuring H_2_O and O_2_ diffusion as well as ROS delivery, is determined by the SiOx intrinsic nanoporosity and its thickness. According to previous studies from our group using advanced in‐depth characterization techniques such as neutron reflectometry, the water hydration of similar superhydrophilic functional layers occurs within seconds.^[^ ^31^ ^]^ Additional morphological characterization such as cross section analysis of np‐SiOx/AgOx/TiOx can be found in S1. Figure 1b.2 shows chemical and (c.2) morphological characterizations of the np‐SiOx plasma polymer. ATR‐FTIR analysis of the thin film in Figure 1b.2 reveals a dominant SiOx chemistry combined with OH groups due to the superhydrophilic behavior of the surface and the Si‐OH pore wall functionalization.^[^ ^30^ ^]^ The SiO_2_ band is shifted to the longitudinal optical (LO) vibrational mode at 1228 cm^−1^, related to the nanoporous nature of the material.^[^ ^32^ ^]^ AFM analysis of the np‐SiOx film in Figure 1c.2 indicates conformal coating by a low average roughness of 0.22 nm. Note that the intrinsic nanoporosity of the plasma polymer results from the oxygen etching of sacrificial hydrocarbons in the silica matrix, yielding a pore size ≤ 1 nm^[^ ^30^ ^]^ and a very low surface roughness. Contact angle measurements showing the hydrophilic and superhydrophilic wettability of AgOx/TiOx and np‐SiOx/AgOx/TiOx, respectively, are included in S2 (Supporting Information).

**Figure 2:**
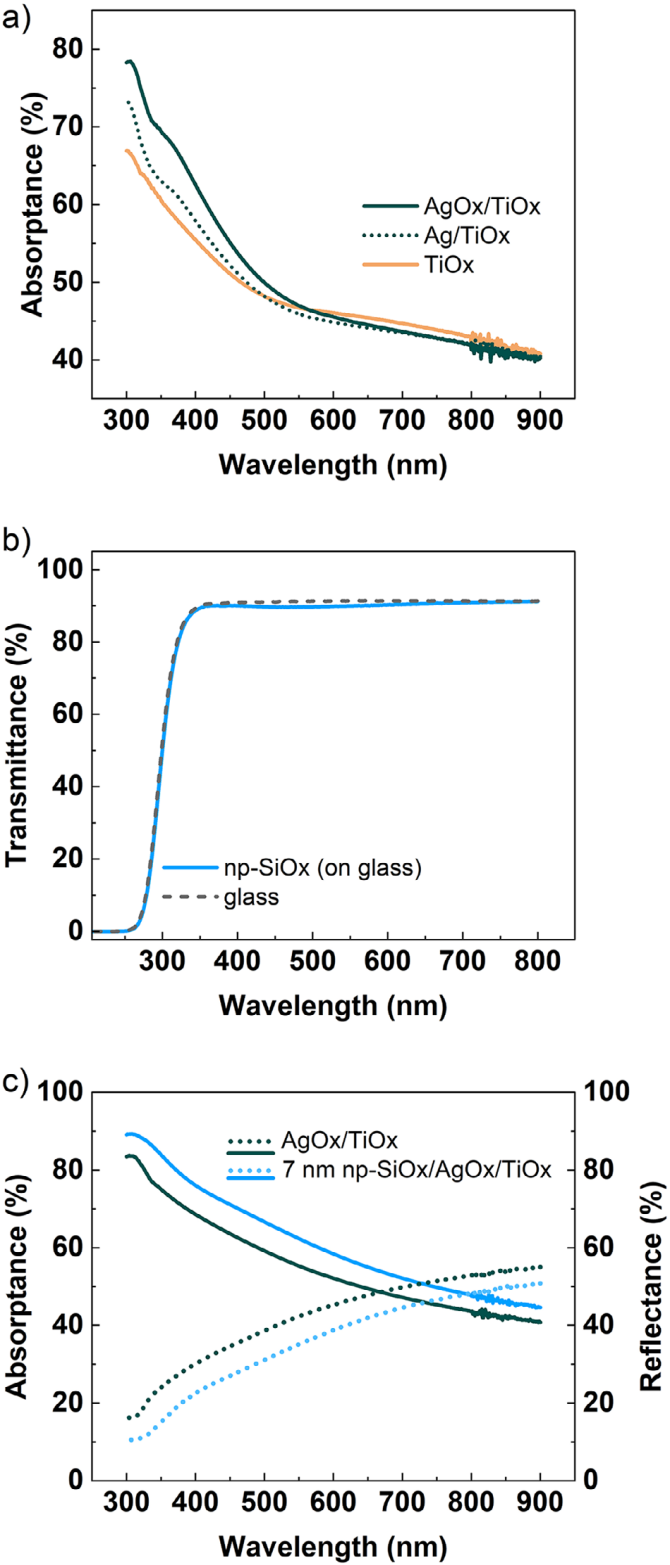
UV–vis characterization of catalytic plasma coatings. a) Absorptance spectra of the different layers of the catalytic metal oxide (TiOx support, Ag/TiOx and AgOx/TiOx; thickness 55 ± 5 nm). b) Similar transmittance of (continuous line) np‐SiOx deposited on glass and (dashed line) glass substrate. c) Comparative (continuous line) absorptance and (dashed line) reflectance spectra of AgOx/TiOx and np‐SiOx/AgOx/TiOx plasma coatings, showing the anti‐reflective behavior of the np‐SiOx layer (7 nm np‐SiOx, 55 ± 5 nm AgOx/TiOx).

Absolute UV–vis optical characterization (300–900 nm, **Figure** **2**) of the catalytic plasma coating and its functionalization was recorded with an integrating sphere. Figure 2a shows the absorptance (i.e., the percentage of light not transmitted nor reflected) of the different layers concerning the fabrication process of AgOx/TiOx. Compared to standard anatase TiO_2_ materials,^[^ ^33^ ^]^ the nonstoichiometric TiOx layer is already characterized by light absorption in the full UV–vis range instead of having a defined band gap or only absorptance in the UV. This is due to the oxygen vacancies introduced during plasma processing,^[^ ^28^ ^]^ retarding also charge recombination.^[^ ^34^ ^]^ In this regard, Figure 2a shows effects of tuning the absorptance by adding Ag and plasma post‐oxidation. Additionally, transmittance measurements of the np‐SiOx layer indicate its transparency with transmittance of ≈90% and no specific absorption bands, overlapping with the transmittance of a glass substrate (see Figure 2b). This result points out that there is no light loss due to the addition of the functional np‐SiOx layer. Indeed, an opposite effect has been detected: Figure 2c gives absorptance and total reflectance curves for an AgOx/TiOx thin film and the same film covered by a 7 nm functional layer (np‐SiOx/AgOx/TiOx). All curves follow the same behavior, indicating no changes in the structure of the band gap, but with an offset positive contribution for the absorption of the functionalized sample with respect to the non‐coated catalyst. Certainly, the np‐SiOx thin layer acts as an anti‐reflective coating,^[^ ^35^ ^]^ as indicated by the opposite tendency observed in total reflectance measurements (see dashed plots in Figure 2c). No differences in total transmittance or the diffuse components were detected, as shown in S3 (Supporting Information). Indeed, this antireflective behavior is expected considering the low refractive index of the np‐SiOx film (*n* = 1.43, according to ellipsometry measurements). A similar optical behavior is expected for thicker functional np‐SiOx layers since the thickness range varied in this study (7–100 nm) is generally below one‐quarter of the wavelength of the incident light.^[^ ^35^ ^]^

Small differences in absorptance behavior can be observed by comparing the AgOx/TiOx curves in Figure 2a,c, respectively. These are due to small differences in thickness in the 55 ± 5 nm range. Nearly negligible values of the spectral diffuse components indicate a high optical quality of the plasma coatings (see S3, Supporting Information). The results reported above demonstrate the transparent and anti‐reflective behavior of the np‐SiOx functional layers. Additionally, their conformal and low‐roughness morphologies, combined with the nanoporous superhydrophilic nature and oxidation‐resistant SiOx chemistry, make them ideal candidates for controlling ROS delivery based on nano‐scaled polymer thickness.

### 2.2 Production and Delivery of ROS by Functionalized Catalytic Plasma Coatings

**Figure 3:**
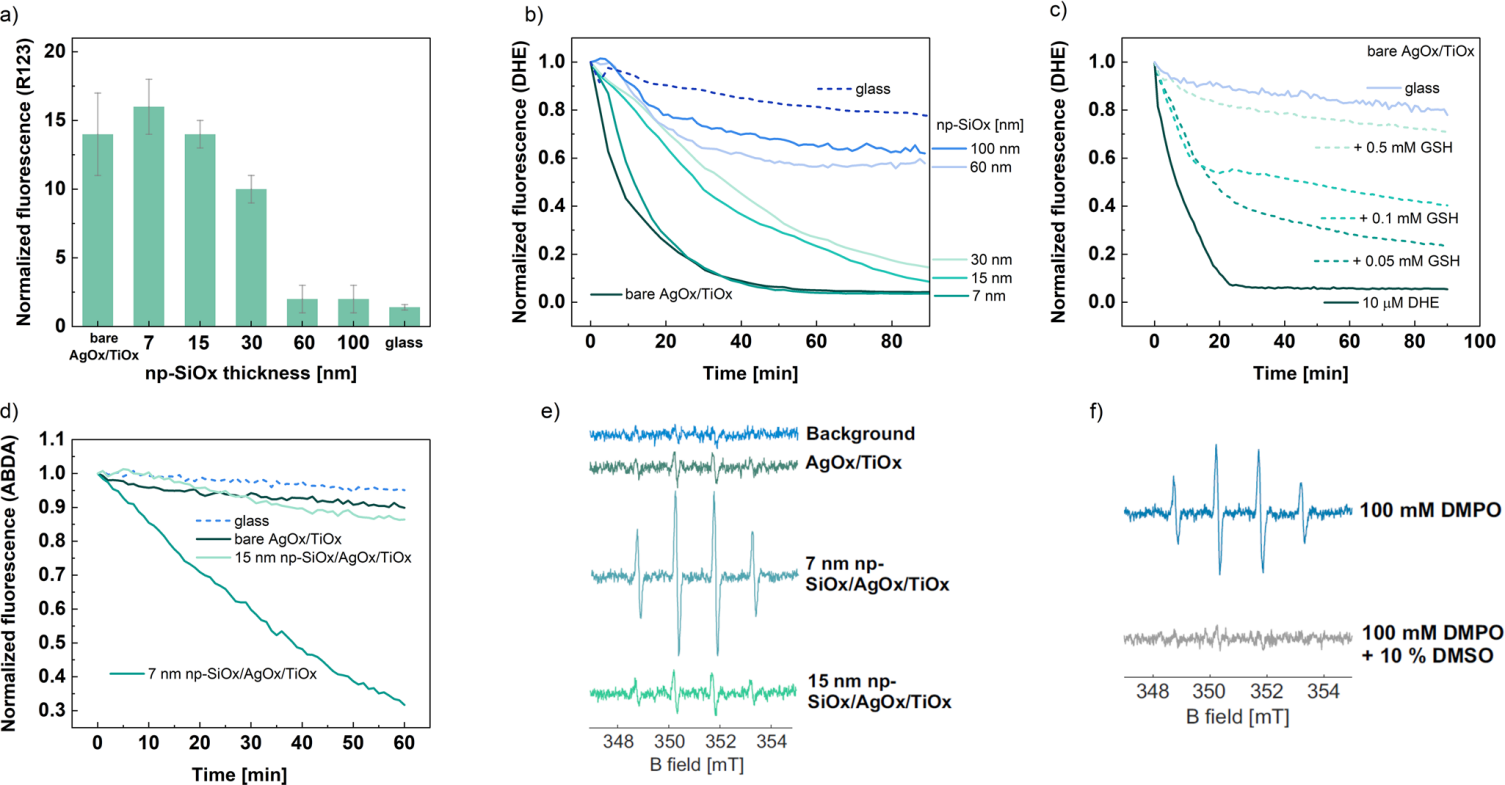
Detection of ROS produced and released by functionalized catalytic plasma coatings by a–d) fluorescence spectroscopy and e–f) EPR. a) General detection of ROS by following R 123 detection at 35 min of incubation (10 µm‐DHR 123 solution). b) Specific detection of O_2_^•–^ by following DHE degradation (10 µm‐DHE solution). c) Scavenging of superoxide with glutathione (GSH) following the DHE degradation (10 µm) with different concentrations of GSH (0.05, 0.1, and 0.5 mm). d) Specific detection of ^1^O_2_ by following the degradation of ABDA (2.4 µm‐ABDA solution). e) Specific detection of •OH radicals by EPR using 100 mm DMPO in 20 mm HEPES (150 mm NaCl buffer, pH 7.3, after 44 min of incubation at RT; additional information can be found in Figure S6, Supporting Information). f) Scavenging of •OH radicals adding 10% DMSO in solution.

In our previous study, the estimation for the ROS production was related to the antibacterial efficacy of the bare AgOx/TiOx material after exposure to daylight.^[^ ^27^ ^]^ Indeed, the UV–vis absorptance characterization (see Figure 2) demonstrates that the catalytic plasma coating absorbs light in the visible range, providing the necessary energy to activate the catalyst, while the nanostructures shown in Figure 1 act as catalytic active sites for the reactions to take place. When H_2_O and O_2_ molecules reach these interfaces, short‐lived ROS are formed (•OH, O_2_^•–^), which can recombine afterward to long‐lived H_2_O_2_. **Figure** **3** presents general and specific detection of different radicals for the AgOx/TiOx catalytic plasma coating and its functionalization with the nanoporous SiOx layer. Overall ROS detection levels are measured by following the ROS‐induced oxidation of dihydrorhodamine 123 (DHR 123) into the fluorescent rhodamine 123 (R 123), shown in Figure 3a. The fluorescence signal is normalized with respect to the control DHR 123 solution. A tendency between the polymer thickness and R 123 detection can be observed, suggesting that the release of radicals is tuned by controlling the thickness of the np‐SiOx layer. The maximum activity is detected for the sample with the thinnest (7 nm) functionalization, then decreasing with increasing np‐SiOx thickness, with no significant R 123 detection for functional layers thicker than 60 nm. In this latter case, the ROS signal is similar to that of the glass (substrate) control. The higher activity of the 7 nm SiOx functionalized sample with respect to the bare catalyst (i.e., bare AgOx/TiOx) can be related to different factors: i) the superhydrophilic (contact angle below 10°) wetting behaviour of the np‐SiOx layer compared to the less hydrophilic AgOx/TiOx surface (≈60°; see S2, Supporting Information), ii) a possible additional activating oxidation effect of the catalyst, during plasma functionalization, slightly affecting its morphology and surface oxidation (see Figure 1a.2) and iii) the higher light absorption shown in Figure 2c. However, the latter point should play a minor role, as fluorescence measurements are carried out in the plate reader (i.e., in the dark), only exposing the material to the 488 nm excitation wavelength for R 123 detection. Moreover, the possibility of a further oxidation of R 123 cannot be discarded, which would avoid its detection, decreasing overall ROS levels. This tendency found between ROS detection and the polymer thickness can be ascribed to the lifetime and the recombination probability of reactive species within the nanoporous SiOx channels. Longer free paths through the polymer matrix may promote ROS recombination at the pores, even for long‐lived H_2_O_2_, finally producing H_2_O and O_2_ and reducing the oxidative activity. Additional measurements of H_2_O_2_ using luminol^[^ ^36^ ^]^ – a specific chemiluminiscence probe – revealed a H_2_O_2_‐production rate of around 10 nmol cm^−2^ s^−1^ for the bare catalyst, which might be maintained for np‐SiOx functionalizations in the 7–30 nm range based on results in Figure 3a. Besides, DHR 123 is a common probe for detecting reactive species, providing a general idea of the oxidative capability of a system, but not specific information about the involved radicals.

In order to shed light on the mechanisms, experiments were performed with dihydroethidium (DHE), a specific probe for O_2_^•–^ detection. Superoxide is an important radical ion formed from the single electron reduction of O_2_ molecules. Kinetic measurements of DHE degradation reveal a clear correlation between the oxidative capability of the system and the SiOx thickness (Figure 3b). In particular, by fitting the curves based on a pseudo‐first order reaction kinetic, a reproducible decrease in the reaction constant is obtained: for a reaction constant (≈1.4 10^−3^ s^−1^) for the AgOx/TiOx plasma coating, it is reduced by 35%, 67%, and 72% for 7, 15, and 30 nm np‐SiOx functionalizations, respectively. For np‐SiOx functional layers thicker than 60 nm, a very small activity is detected, with a kinetic comparable to that of the glass substrate, similar to what has been shown for DHR 123 (Figure 3a). Additional details regarding DHE kinetic degradation can be found in S4 (Supporting Information).

To confirm that superoxide was generated by the reduction of O_2_, the same experiment was performed in anoxic (no O_2_) conditions (see S5, Supporting Information for details). In these conditions, the drop in oxidation efficiency of the catalytic plasma coating confirms that the process is O_2_‐mediated. Moreover, the production of superoxide radicals was confirmed with an inhibition (i.e., scavenging) experiment performed with glutathione (GSH, see Figure 3c). GSH is a major non‐enzymatic ROS scavenger preferentially oxidized in presence of O_2_^•–^; this antioxidant property plays a key role in protecting mammalian cells from ROS‐induced oxidative damage.^[^ ^37^ ^]^ Therefore, when added to a DHE solution, GSH can compete with the dye to be oxidized by O_2_^•–^. A clear inhibition of DHE degradation is observed for GSH concentrations more than ten times higher than that of DHE, confirming that that O_2_^•–^ is generated and can be scavenged by GSH. Overall, our results indicate that superoxide is formed from reduction of molecular oxygen and that their delivery can be controlled by the thickness of the np‐SiOx layer. In terms of superoxide production, the higher concentrations are detected for the AgOx/TiOx and 7 nm np‐SiOx/AgOx/TiOx systems, able to fully oxidize DHE in one hour. Taking into account that the concentration of the fluorescent probe was 10 µm in 300 µL (i.e., 3 nmol), that the diameter of the samples was 1 cm (i.e., surface of 0.785 cm^2^), and the stochiometry between O_2_^•–^ and DHE is 1:1, we can conclude that the production of superoxide by active plasma coatings is at least 0.11 nmol cm^−2^ s^−1^ in normoxic conditions. Based on the GSH inhibition experiment, this production rate may be more than ten times higher. We can thus confirm that a strong superoxide formation efficiency is present, with kinetics similar to other photocatalytic systems,^[^ ^38^ ^]^ but without the need for light stimuli during the experiment. As mentioned above, the catalyst is active after light exposure, but the analyzed fluorescence experiments were performed in dark conditions, with notable catalytic activity maintained due to charge separation.

To further investigate the type of ROS produced, another specific fluorescent probe has been used. Figure 3d presents an experiment carried out with a 9,10‐Anthracenediyl‐bis(methylene)dimalonic acid (ABDA) solution. ABDA is a probe that is specifically oxydized by singlet oxygen (^1^O_2_) into a non‐fluorescent product.^[^ ^39^ ^]^ Therefore, singlet oxygen production was monitored by following the decrease in fluorescence intensity.^[^ ^40^ ^]^ The AgOx/TiOx sample does not result in a loss in fluorescence, with similar behavior as the glass substrate, revealing there is no ^1^O_2_ production by the catalytic plasma coatings. Surprinsingly, a clear decrease in the fluorescence signal is detected only for the sample with the thinnest functional polymer (i.e., 7 nm np‐SiOx/AgOx/TiOx), which is also in agreement with the highest overall ROS detection by DHR in Figure 3a, without measured decrease for thicker functional layers (i.e., *d*_np‐SiOx_ > 7 nm). For photocatalytic TiO_2_ materials in aqueous suspensions, the main mechanism responsible for ^1^O_2_ production is considered to be O_2_^•–^ oxidation at the holes.^[^ ^41^, ^42^ ^]^ We propose that the addition of the nanoporous functional layer increases the residence time of superoxide radicals at the catalyst surface, promoting O_2_^•–^ to ^1^O_2_ oxidation. Specific ^1^O_2_ formation has also been reported by Parrino et al. for silanized TiO_2_ nanoparticles, with no ^1^O_2_ detection without adding the functional organosilicon groups.^[^ ^22^ ^]^ This O_2_^•–^ to ^1^O_2_ conversion might similarly happen for thicker plasma polymer functionalization, although ^1^O_2_ is not detected for *d*_np‐SiOx_ > 7 nm, probably due to their recombination within the thicker nanoporous matrix before reaching the external aqueous phase. Indeed, singlet oxygen is characterized by a shorter lifetime in water (microseconds at 25 °C),^[^ ^43^ ^]^ compared to other ROS under similar conditions such as O_2_^•–^ (miliseconds)^[^ ^44^ ^]^ or H_2_O_2_ (hours to days).^[^ ^45^ ^]^ Therefore, very thin plasma polymer functionalization allows delivery of ^1^O_2_, specifically tunning the type of ROS produced. This fact opens the room for applications when applying ultra‐thin nano‐scaled functionalization, with singlet oxygen as a desired reactive species due to its strong oxidative properties.^[^ ^43^ ^]^

It is important to remark that the used fluorescent probes, i.e., DHR 123, DHE, and ABDA, are considerably large molecules with several aromatic rings. They are too large (>1 nm) to penetrate and diffuse with H_2_O through the nanoporous network (≤1 nm). Hence, they are not directly oxidized by the active surface (i.e., the AgOx/TiOx surface). ROS measured by fluorescence spectroscopy are detected outside the nanoporous plasma polymer, demonstrating the ROS‐delivery capability of plasma functionalization. A different situation occurs for the following experiment aiming to detect •OH radicals. Figure 3e shows continuous wave electron paramagnetic resonance (cw EPR) spectra with 5,5‐Dimethyl‐1‐pyrroline *N*‐oxide (DMPO) present in solution as a spin trap for ROS. The presence of hydroxyl radicals is detected for both AgOx/TiOx and np‐SiOx/AgOx/TiOx systems. After 44 min of incubation, the highest signal is obtained for the 7 nm np‐SiOx/AgOx/TiOx sample, while similar overall intensities are obtained for the bare catalyst and thicker functionalizations (see S6 for complementary data). It is important to remark that, in this case, to accelerate the ROS production reaction kinetic, experiments were performed while incubating the sample under light exposure (common LED lamp). To confirm that the DMPO**^·^**‐OH signals arise from direct trapping of •OH by DMPO, we added DMSO to the aqueous solution, which is a known scavenger of •OH radicals. After prolonged incubation in presence of DMSO, we found a negligible DMPO**^·^**‐OH signal, proving that the EPR signals detected in aqueous buffers (Figure 3e) specifically probe the formation of •OH radicals and are not caused by degradation of short‐lived DMPO·‐OOH adducts into DMPO·‐OH (see Figure 3f). In contrast to DHR 123, DHE, and ABDA, the DMPO molecule is much smaller in size, enabling it to traverse through the porous network and reach the AgOx/TiOx surface where catalytic reactions are taking place. For this reason, •OH radicals are detected for all the tested functionalized samples regardless of thickness (7, 15, and 30 nm np‐SiOx). Otherwise, they should not have been detected, taking into account the short lifetime of these radicals (nanoseconds), generally scavenged a few angstroms from its generation site.^[^ ^46^ ^]^ The enhanced detection of •OH radicals with DMPO by EPR (see Figure 3e) should trigger a higher concentration of H_2_O_2_ for the 7 nm np‐SiOx functionalization compared to the plain catalyst (AgOx/TiOx). This, together with the singlet oxygen detection shown in Figure 3d, agrees with the higher overall ROS detection by following the oxidation of DHR 123 (see Figure 3a) for the 7 nm functionalization.

**Scheme 2:**
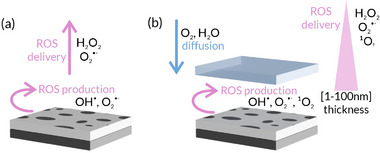
ROS production (AgOx/TiOx) and controlled delivery (np‐SiOx/AgOx/TiOx) based on the thickness of the functional nanoporous layer.

The mechanisms of O_2_^•–^ and •OH radical generation involve electrons and holes separated in the catalyst, where electrons reduce electron acceptors (molecular oxygen, yielding O_2_^•–^), and holes oxidize electron donors including adsorbed water (H_2_O) and hydroxides (OH^–^) to give hydroxyl (•OH) radicals.^[^ ^47^, ^48^ ^]^ Generally, O_2_^•–^ and •OH radicals can further react to yield H_2_O_2_, eventually forming H_2_O and O_2_. **Scheme** **2** illustrates the mechanistic view from Figure 3, with ROS detection (H for the bare AgOx/TiOx catalyst and controlled ROS delivery when including the functional layer (np‐SiOx/AgOx/TiOx). Precise control of the np‐SiOx thickness allows to specifically deliver ^1^O_2_ as well as the controlled release of longer‐lived reactive species. This capability of controlling ROS delivery represents a promising strategy for therapeutic applications. It goes a step further compared to other approaches such as the use of ROS gels for a slow release over an extended period of time,^[^ ^3^ ^]^ enabling sustained continuous release of ROS to a target site. Taking into account the nanoporosity of the SiOx layer and its superhydrophilic surface, water and oxygen need only seconds to penetrate and hydrate the layer (see right sketch in Scheme 2).^[^ ^31^ ^]^ Results obtained with the added np‐SiOx layer indicate that the accesibility of the catalytic active sites is maintained at the covered interface. Once produced at the interface, ROS diffuse to the outside through the nanoporous network, while they might recombine among themselves and with ‐OH groups at the pore walls (functionalized with Si‐OH groups).^[^ ^30^ ^]^ For np‐SiOx layers thicker than 30 nm, O_2_^•–^ is no longer detected. This suggests that most short‐lived radicals likely recombined within the nanoporous matrix, leading to the formation H_2_O_2_ but also H_2_O and O_2_. Given the small size of DMPO molecules, it is not possible to assess •OH delivery, although these radicals should recombine within the first few nanometers. As mentioned, the thin np‐SiOx functionalization of 7 nm allows to specifically deliver ^1^O_2_. We performed further measurements to assess the role of the porous volume of the plasma functionalization. Comparative ROS values were detected for np‐SiOx layers with a porous volume of 13 ± 2% and 20 ± 5%. These negligible differences indicate that the layer thickness is the rate‐limiting step rather than the porous volume of the coating.

### 2.3 Production and Delivery of ROS below Cytotoxicity and Sensitization Levels

**Figure 4:**
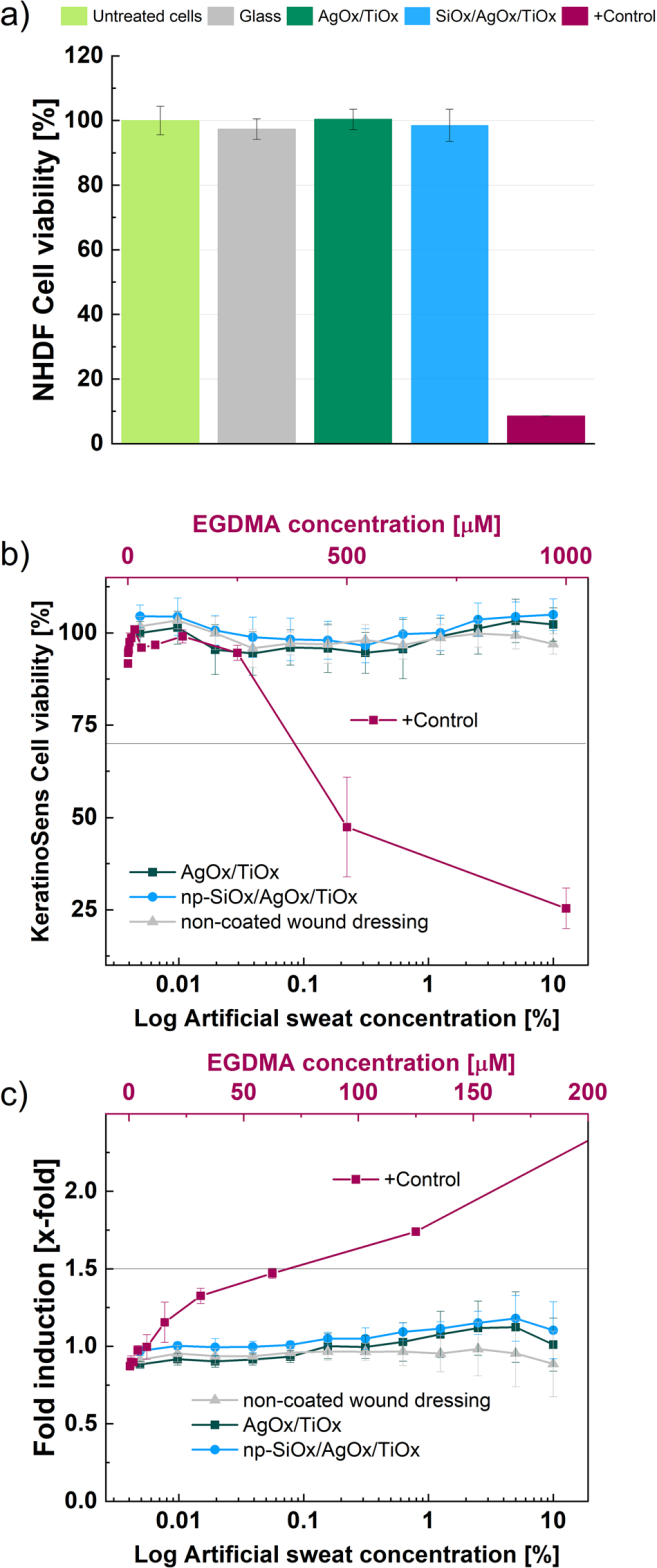
a) Cytotoxicity tested with NHDF using MTS assay, b) KeratinoSens^TM^ skin irritation (cell viability) and c) sensitization (fold induction) assay. The np‐SiOx layer has a 15 nm thickness for both tests. Positive controls (i.e., +Control) are a) 1% Triton X‐100 and b,c) EGDMA. Substrates are also tested, as negative controls, a) glass and b,c) non‐coated wound dressing. N = 3.

Focusing on biological and medical applications, one key aspect for a ROS‐releasing platform is the absence of cell cytotoxicity. Similar requirements can be expected for further applications where the catalytic material may be in contact with ecosystems. The AgOx/TiOx material exhibited no cytotoxicity itself after an incubation time of 24 h, as confirmed by the results in **Figure** **4**a, using normal human dermal fibroblasts (NHDFs, C‐12352, PromoCell) following the ISO10993‐5 norm. Additionally, the effect of adding the np‐SiOx functional layer was found to induce no cytotoxicity. A positive control consisting of a 1% Triton X‐100 solution in DMEM is included in Figure 4a to properly control cell viability. Furthermore, to test the potential of the catalytic plasma coating for skin treatments, cell viability and sensitization have been tested in artificial sweat also for 24 h in contact. For these tests, the catalytic plasma coatings are directly deposited on wound dressings as shown in the photograph in Scheme 1. This test is primarily designed to study the release of ions in an artificial sweat solution, an important factor to consider for nanomaterials composed of metal oxides as in the present case. In our previous publication, for an incubation time of 24 h, no significant release of Ag ions is detected (below 0.1 µg cm ^2^).^[^ ^27^ ^]^ In agreement with this previous characterization, results reported in Figure 4b,c show no effect on skin cell viability or sensitization (fold induction) according to OECD guideline 442D, respectively, for both AgOx/TiOx and np‐SiOx/AgOx/TiOx samples. To confirm the accuracy of the test, positive controls consisting of ethylene glycol dimethacrylate (EGDMA, cell toxic and sensitizer) indicate a high power of sensitizing and irritation. Conversely, no adverse events for advanced skin cultures are expected for the ROS‐releasing platform. This makes the plasma coatings suitable for biomedical applications, such as treating skin infections or coating external surfaces that may come into contact with living organisms (e.g., face masks, surgical instruments). These applications also benefit from the coatings' long‐term stability, as demonstrated by our previous results showing sustained catalytic activity even years after fabrication.^[^ ^27^ ^]^

### 2.4 ROS‐Correlated Antibacterial and Antiviral Activity of Functionalized Catalytic Plasma Coatings

**Figure 5:**
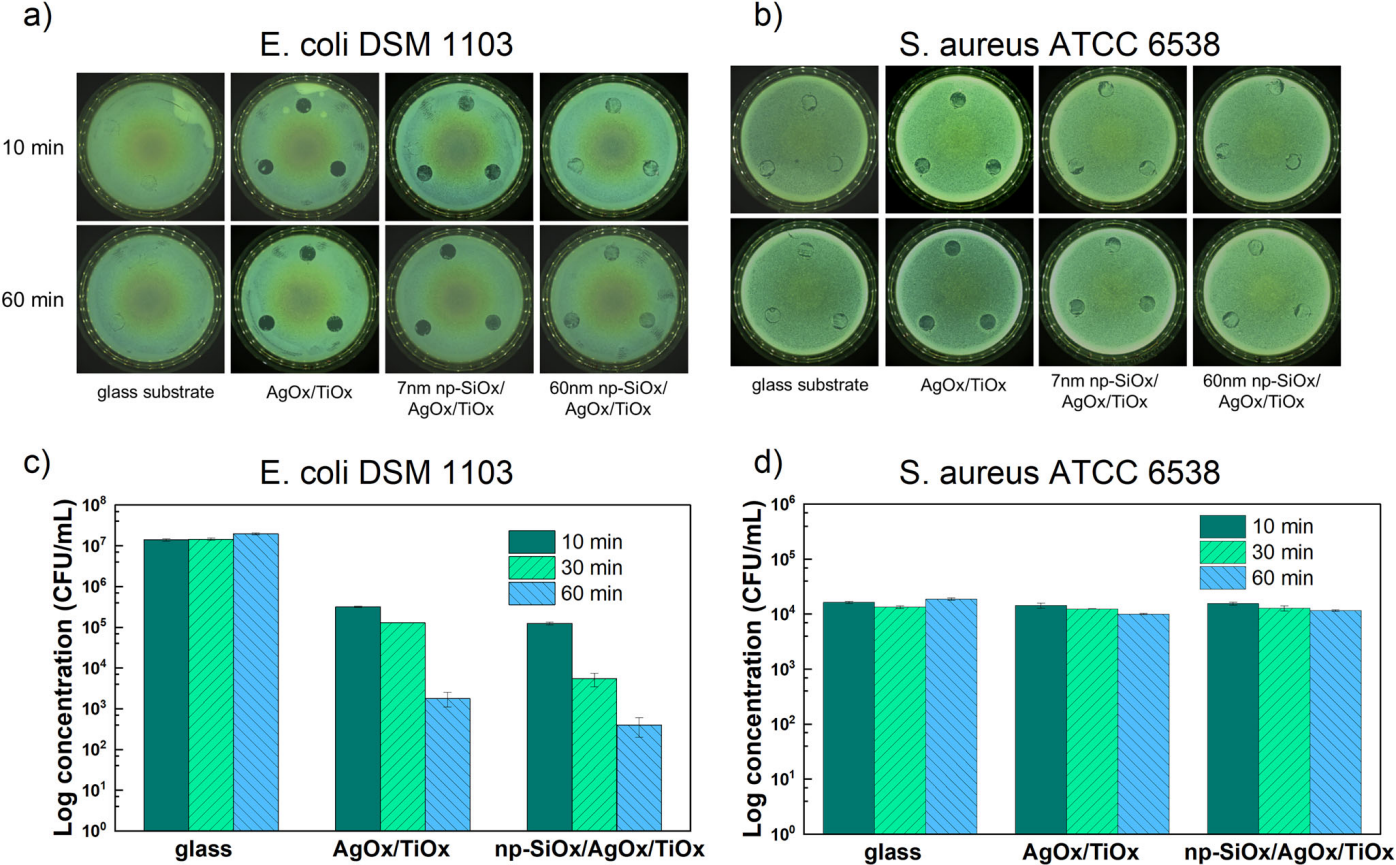
Antibacterial activity of the catalytic plasma coatings. a,b) Qualitative analysis of the antibacterial property of AgOx/TiOx and the functionalization with 7 and 60 nm np‐SiOx against a) E. coli DSM 1103 and b) S. aureus ATCC 6538, for 10 and 60 min of incubation. c,d) Quantitative analysis of the antibacterial property of AgOx/TiOx and a functionalization with an intermediate thickness of 15 nm for c) E. coli DSM 1103 and d) S. aureus ATCC 6538 at three different contact times of 10, 30, and 60 min. N = 3.

To demonstrate a potential application, the detection of radicals has been associated with the antimicrobial activity of the ROS‐releasing system. First, the catalytic plasma coatings were qualitatively analyzed for their antibacterial activity aginst the gram‐negative strain *E. coli* (**Figure** **5**a) and the gram‐positive strain *S. aureus* (Figure 5b) using a touch test. As can be observed in the figure, the negative control glass substrate did not impact the growth. Against *E. coli*, both the AgOx/TiOx and 7 nm np‐SiOx/AgOx/TiOx samples revealed clear growth inhibition already after 10 min, with further enhancement observed after 60 min. A minor inhibition compared to the glass negative control is detected for the sample functionalized with a thicker (60 nm) np‐SiOx coating, slightly increasing after 60 min. A generally higher activity is inferred for the plain catalyst, indicating a slightly delayed activity when the thin plasma polymer is added. The data obtained here are consistent with those discussed above, i.e., thicker (>60 nm) np‐SiOx layers hindering ROS delivery (Figure 3) ultimately resulting in limited bacterial inhibition. Against *S. aureus* (Figure 5b), the antibacterial activity of the coatings is less pronounced, which aligns with earlier findings^[^ ^27^, ^49^ ^]^ related to the inherent resistance of Gram‐positive bacteria due to their thicker peptidoglycan layer.^[^ ^49^, ^50^ ^]^ Nevertheless, the same tendency in inhibition as for *E. coli* (c.f. Figure 5a) is detected: a stronger effect after 60 min compared to 10 min of interaction, particularly for the 7 nm np‐SiOx/AgOx/TiOx sample.

Second, to quantitatively analyze the antibacterial activity, a modified ASTM E2180 method was employed to assess the efficacy against the same bacterial strains. Figure 5c,d presents the obtained results for different exposure times (10, 30, and 60 min) for the AgOx/TiOx plasma coating and one functionalization thickness of 15 nm np‐SiOx. This intermediate thickness has been selected with the aim of quantitatively detecting a delay in the antibacterial properties of the system. For *E. coli* (Figure 5c), the functionalized sample seems to be similarly active as the bare catalyst. Note that superhydrophilic surfaces can affect bacteria adhesion because the water layer acts as a barrier,^[^ ^51^ ^]^ which might counterpart a lower dose of ROS. As can be observed in Figure 5c, after 10 min, both coatings demonstrate similar antibacterial efficacy of 2 logs reduction in colony‐forming units (CFU). Extending the interaction time, CFU further reduce, reaching after 60 min over four logs of reduction for the functionalized sample and slightly less than four logs for the AgOx/TiOx samples. These results indicate that sufficient ROS generation is achieved for effective *E. coli* inhibition in both the AgOx/TiOx and np‐SiOx/AgOx/TiOx systems. On the contrary, almost no activity is detected for *S. aureus* by this method, as shown in Figure 5d, for both bare and functionalized catalytic plasma coatings. *S. aureus* (Gram‐positive bacteria) is generally considered more resistant to ROS than *E. coli* (Gram‐negative).^[^ ^50^ ^]^ This increased resistance can be attributed to several well‐characterized mechanisms possessed by *S. aureus*. For instance, this strain produces carotenoid pigments (notably staphyloxanthin), which act as potent antioxidants by quenching ROS. In contrast, E. coli relies on enterobactin, which is less effective in scavenging ROS.^[^ ^52^ ^]^ Furthermore, S. aureus produces multiple enzymes, such as catalases, peroxiredoxins, and other peroxidases, to help detoxify hydrogen peroxide and organic peroxides.^[^ ^50^ ^]^ E.g., *S. aureus* demonstrates 30–50% higher catalase activity than *E. coli* under oxidative stress, enabling more efficient detoxification of hydrogen peroxide and other peroxides. This enzymatic defense may reduce the net ROS concentration reaching critical intracellular targets in shorter‐duration assays, requiring higher local ROS concentrations or longer time for inactivation.^[^ ^49^, ^50^ ^]^ *E. coli* does have its own ROS‐detoxifying systems (e.g., OxyR regulon, catalases, peroxidases), but its overall resistance is less robust compared to *S. aureus*.^[^ ^50^, ^53^ ^]^ Finally, *S. aureus*, as a Gram‐positive bacterium, possesses a thick peptidoglycan layer (20–80 nm), which acts as a physical barrier to ROS penetration. In contrast, *E. coli* has a thinner peptidoglycan layer (2–3 nm), making it more vulnerable to ROS.^[^ ^49^ ^]^ Applying these insights to the present study, the lack of inhibition observed for *S. aureus* with the 7 nm np‐SiOx coating for up to 60 min could be explained by insufficient ROS accumulation or inadequate interaction time to surpass the structural defenses of *S. aureus*.

**Figure 6:**
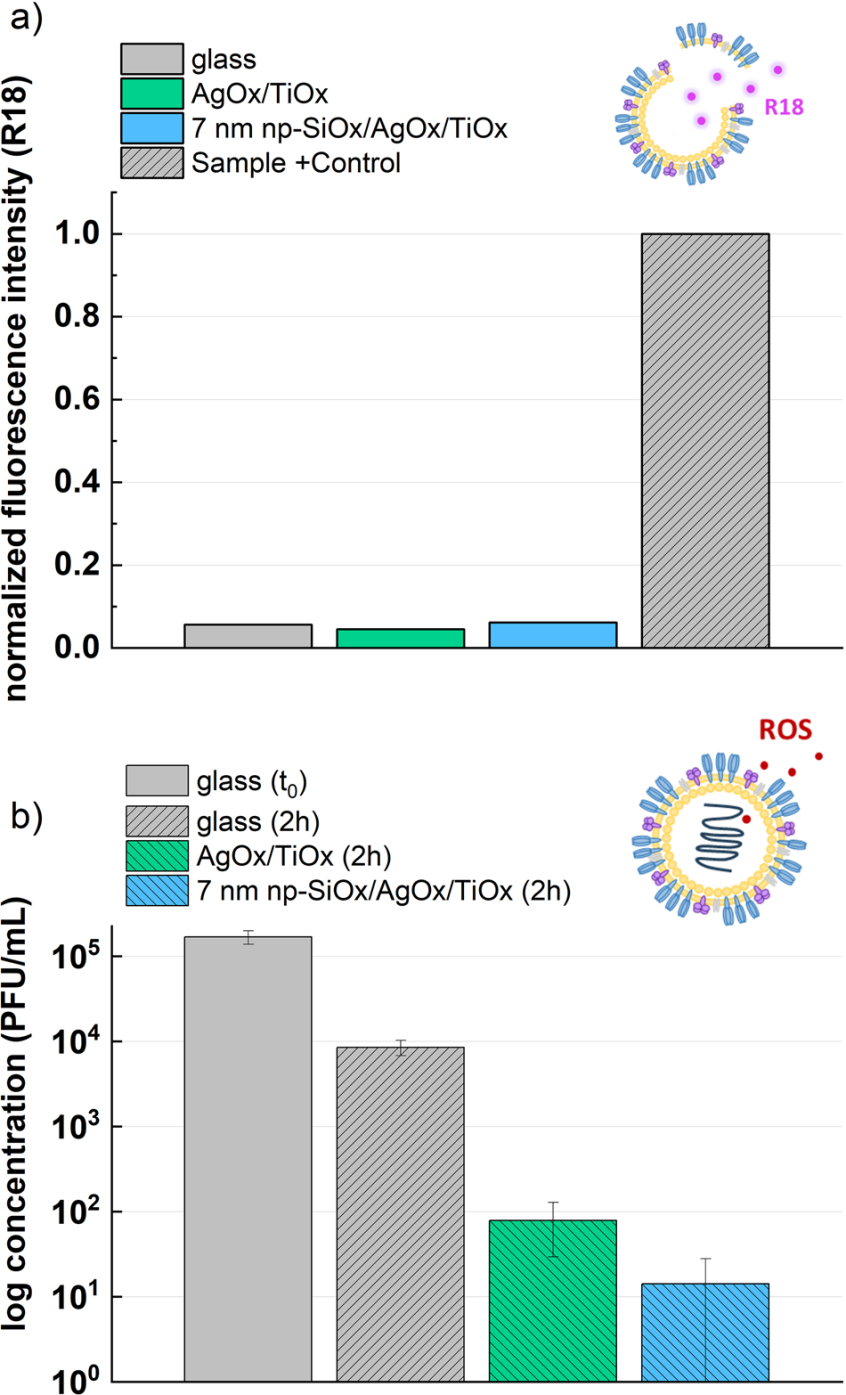
Antiviral tests for AgOx/TiOx and 7 nm np‐SiOx/AgOx/TiOx plasma coatings. a) InVIS rhodamine‐18‐quenched virus experiment, for each sample including a positive control (adding an OEG detergent). b) MHV inhibition performed according to ISO 18184 plaque assay. Glass substrates were used as negative control. N = 3.

The antiviral properties of AgOx/TiOx and functionalized np‐SiOx/AgOx/TiOx plasma coatings have been also investigated by using two different tests regarding the interaction between the produced ROS and two virus models. **Figure** **6**a gives results for an InVIS assay.^[^ ^54^ ^]^ This test involves quenching a fluorescent rhodamine‐18 probe within the viral membrane of an inactivated Brisbane/2007 influenza virus model. When the virus envelope is intact, no fluorescence is detected, but upon disintegration of the viral capsule, the fluorescence signal of the rhodamine can be measured. AgOx/TiOx and 7 nm np‐SiOx/AgOx/TiOx plasma coatings and a glass substrate were tested, the latter included as negative control. For positive controls, the incubation of the virus, together with the samples, is mixed with an octaethylene glycol monododecyl ether OEG detergent that disintegrates the virus by membrane dissolution and protein denaturation.^[^ ^54^ ^]^

As can be observed in Figure 6a, only a low baseline fluorescence signal is detected for both the glass, AgOx/TiOx, and 7 nm np‐SiOx/AgOx/TiOx plasma coatings, indicating that the virus membrane remains intact, whereas adding the detergent to all samples induces the charasteristic fluorescence due to viral envelope disintegration. The release of metal ions such as copper ions^[^ ^55^ ^]^ or the sharp edged structure of graphene oxide^[^ ^56^ ^]^ has been claimed as possible agents to affect the viral capsid integrity.^[^ The absence of ion release and the low, smooth surface roughness of the plasma coatings justify the results in Figure 6a.

Nevertheless, as for bacteria, there are different possible mechanisms responsible of virus inactivation and destruction aside membrane breakage such as reactions with targeted proteins^[^ ^57^ ^]^ and ROS diffusion thorugh the membrane.^[^ ^58^ ^]^ Indeed, 2 and 3 logs of reduction of virus concentration can be seen in Figure 6b for AgOx/TiOx and 7 nm np‐SiOx/AgOx/TiOx samples, respectively, after two hours of incubation of an active murine hepatitis virus (MHV) according to ISO 18184 with respect to the negative control (i.e., glass substrate after 2 h). It is relevant to study comparable times of incubation, since the viability of a virus on a surface is reduced over time.^[^ ^59^ ^]^

Certainly, performing this test for materials deposited on wound dressings, as in Figure 4b,c, gives no significant virus concentration on both substrates (i.e., dressings) and coated dressings after two hours. This is due to the unstable behavior of viruses on textiles, thus ideal substrate materials for antiviral properties.^[^ ^60^ ^]^ Therefore, results in Figure 6b are tested for catalytic plasma coatings deposited on glass, to ensure that the concentration reduction after two hours is due to the material's activity (i.e., ROS release). High antiviral activity is detected for functionalized and non‐functionalized samples, higher in the former case due to the higher general ROS detection for the 7 nm np‐SiOx functionalization and the specific delivery of ^1^O_2_. Indeed, singlet oxygen has been reported as a major oxidizing agent for viral capsid proteins.^[^ ^61^ ^]^ Hence, the antiviral efficacy of the material is demonstrated as a result of ROS diffusion through the virus envelope (i.e., lipid membrane) and the reaction with capsid proteins and DNA^[^ ^57^ ^]^ rather than breaking the virus membrane.

## 3 Conclusions

Plasma surface functionalization with thin, nanoporous SiOx‐like PPFs can be used to control the amount and type of ROS delivered by catalytic plasma coatings. An optimized combination of AgOx and TiOx fabricated by magnetron sputtering at RT is used as a model system of ROS production, working also in the dark (after light exposure), thanks to charge separation. Results indicate that •OH and O_2_^•–^ radicals are primarily formed at the catalytic active surface, followed by their recombination. ROS delivery is precisely controlled at the nanoscale by functionalization with nanoporous, superhydrophilic plasma polymer layers deposited from HMDSO plasma combined with oxygen etching. By varying the thickness of the np‐SiOx layer from 7 to 100 nm, a consistent and specific control of the type and amount of ROS delivered can be achieved. The addition of a very thin (d_np‐SiOx_ < 10 nm) functional layer allows the partial conversion of O_2_^•–^ to ^1^O_2_ and subsequent delivery of both radicals to the outside environment. ROS delivery levels are always below cytotoxicity thresholds, making the material suitable for biomedical applications as well as other contact‐based approaches without causing harm to ecosystems. Additionally, the functional layer avoids direct contact of cells and other substances with the metal oxide surface and ensures the material activity is due to ROS‐driven processes and not by other direct redox chemistry or leaching processes. Results obtained for the production and delivery of ROS have been correlated with the antibacterial and antiviral properties of the catalytic material. Different bacteria and virus models have been studied, indicating that antimicrobial mechanisms are related to ROS diffusion and reactions with key macromolecules of the microorganisms. Hence, a catalytic metal oxide plasma coating combined with plasma polymer functionalization offers a powerful toolbox to provide tailored ROS‐delivering surfaces. Industrial implementation is facilitated based on established plasma technology and one‐step processing.

## 4 Experimental Section

### Fabrication of Catalytic Thin Films (AgOx/TiOx)

The complete fabrication process was carried out in a pilot‐scale plasma chamber equipped with a rotatable drum. The reactor contains separated radiofrequency (RF) plasma and sputtering regions, allowing for the performance of a one‐step process. Silicon wafers, round glass slides, fabrics, and wound dressings were used as substrates. Substrates were cleaned with Ar plasma (160 sccm Ar at 0.1 mbar, 400 W RF‐power for 10 min). After that, first titanium (Ti) and then silver (Ag) targets, located one in front of the other, were sputtered (in the metallic mode). Specifically, a layer of 50 nm of TiOx was sputtered from the Ti target (70 sccm Ar at 0.008 mbar, applying pulsed DC (100 kHz) – 2 kW at 320 V‐ for 8 min). Note that, although no reactive sputtering was carried out (no O_2_ added to the plasma), nonstoichiometric TiOx was formed because of residual water and oxygen content in the chamber.^[^ ^27^, ^62^ ^]^ Subsequently, a nominally 5‐nm‐thick layer of Ag was sputtered from the Ag‐target (70 sccm Ar at 0.008 mbar, applying pulsed DC (100 kHz) – 0.6 kW at 360 V‐ for 36 s). As the deposited Ag is in metallic state, plasma post‐oxidation is applied to oxidize to AgOx. Consequently, samples were exposed to an Ar/O_2_ discharge in the RF‐plasma zone (200 sccm Ar/200 sccm O_2_ at 0.25 mbar, 600 W for 10 min), resulting in AgOx/TiOx with a total thickness of 55 ± 5 nm. A similar fabrication procedure is described by Hegemann et al.^[^ ^27^ ^]^

### Functionalization of Catalytic Thin Films with Nanoporous SiOx Plasma Polymer Films (np‐SiOx/AgOx/TiOx)

AgOx/TiOx catalytic thin films were further coated with nanoporous SiOx (np‐SiOx) PPFs acting as functional layers to precisely control ROS delivery. Functional layers with 13 ± 2% volumetric porosity were fabricated in a symmetric RF capacitively coupled (CCP) plasma chamber, with the samples located at the electrode and fully exposed to the plasma.^[^ ^30^ ^]^ Cycles of plasma polymerization (Ar/O_2_/HMDSO – depositing SiO:CH) and etching (Ar/O_2_ – removing the residual hydrocarbon content over 5 min) were carried out, varying the coating thickness between 7 and 100 nm. Similar layers with a higher volumetric porosity (20 ± 5%) were also fabricated following a similar procedure in the sample plasma chamber used for the fabrication of the AgOx/TiOx coatings. In this case, the samples were located at the wall of the reactor and coated following a method to reduce ion bombardment (near‐plasma chemistry approach).^[^ ^32^ ^]^

### Characterization of AgOx/TiOx and np‐SiOx Thin Films

P‐doped silicon wafers were used as substrates for profilometry and morphological characterization. The thickness of the deposited layers was measured using a Bruker Dektak XT profilometer. SEM micrographs were acquired in top view with a Hitachi S4800 microscope operated at 2 kV detecting secondary electrons. EDS and FIB‐SEM analysis were carried out with a TFS Helios 600i device operated at 3 kV. AFM analysis was conducted using a Bruker Dimension Icon AFM (Billerica, US). The AFM was operated in tapping mode with an Olympus OMCL‐AC160TS‐R3 probe, characterized by a resonant frequency of 300 kHz, a spring constant of 26 N m ^1^, and a nominal tip radius of 7 nm. The obtained images were processed and analyzed using NanoScopeAnalysis software (version 3.00). A polynomial flatten filter (*n* = 1) and a plane fit (polynomial *n* = 1, XY mode) were applied to all presented AFM images. ATR‐FTIR (Varian 640‐IR, Agilent) was used to characterize the chemistry of the functional layers (i.e., PPFs), with the samples deposited on aluminum foil. Optical characterization of the polymers was carried out in a UV–vis Cary 4000 (Agilent) spectrometer. Ellipsometry was used to determine the refractive index and, therefore, the porosity of PPFs, using a Nanofilm EP4 device (Accurion); the device was operated at a constant wavelength of 658 nm and varying the angle of incidence between 55 ° and 80 ° (1° step). For a more detailed explanation see the previous publications.^[^ ^30^, ^32^ ^]^

### ROS Detection by Fluorescence

The production of ROS was monitored in a Biotek Synergy H1 Multimode Reader (Agilent), using specific fluorescence excitation and emission wavelengths. The instrument was operated at normal speed, with a delay of 100 ms and measuring from top. The measurements were conducted by introducing the samples deposited on glass (10 mm diameter) in a 48 well‐plate and pipetting 300 µL of water‐based solutions of the specific dyes. The reproducibility of the results was checked also working with a 96‐well plate, 6 mm samples and pipetting 100 µL solutions. The signal of the glass substrate, as well as the dye itself, was controlled during the measurements. The following molecules were used for the detection: dihydrorhodamin 123 (DHR 123, Sigma Aldrich), at concentration 10 µm, following its oxidation into rhodamine 123 with excitation at 488 nm and emission at 535 nm; dihydroethidium (DHE, Sigma Aldrich), at concentration 10 µm, following the degradation of the dye itself with excitation at 355 nm and emission at 420 nm; 9,10‐Anthracenediyl‐bis(methylene)dimalonic acid (ABDA), at concentration 2.4 µm, with excitation at 404 nm and emission at 490 nm. To ensure reproducibility, experiments were conducted more than three times, with a minimum of three replicates per experiment.

Additionally, the production rate of H_2_O_2_ was evaluated using a luminol‐based chemiluminescence protocol,^[^ ^36^ ^]^ with measurements performed on the same instrument. The probe solution was prepared by mixing iron(III) chloride hexahydrate (27 g L ^1^) and luminol (75 g L ^1^) in a buffer solution (pH 11.87), diluted in a ratio of 1:10:100, respectively, resulting in a final luminol concentration of 50 µm. A calibration curve for H_2_O_2_ in the range of 0.5 to 2 mm was used to obtain approximate quantitative data. For each measurement, 50 µL droplets of the probe solution were deposited onto the glass‐coated samples placed in a 24‐well black plate. Chemiluminescence was recorded immediately after contact, with signal integration over 5 s.

### ROS Detection by Spin Trapping EPR

Catalytic plasma coatings were incubated in a 24‐well plate with 200 µL of 100 mm DMPO (Dojindo) in 20 mm HEPES, 150 mm NaCl buffer at pH 7.3, under continuous light irradiation with an LED lamp (3.4 W, 220 V). All the kinetics of DMPO**^·^**‐OH adduct formation were performed at RT. Approximately every 10 min (with an offset of 3 min for the first point), 20 µL of solution were collected in a glass tube of 0.7 mm i.d. and measured with a Bruker E500 X‐band cw EPR spectrometer, coupled with an ER 4122 SHQ cavity. For each measurement, 80 G were swept in 60 s with a time constant of 40.96 ms at a microwave power of 9.85 mW. The modulation was set to 1.5 G of amplitude and 100 kHz of frequency. For each measurement, four scans were averaged, and then the sample was recollected from the glass tube and pipetted back in the well plate.

### Cytotoxicity

Possible cytotoxicity of the catalytic plasma coatings was analyzed using NHDFs (C‐12352; PromoCell). Samples were extracted in 150 µL DMEM (Dulbecco's Modified Eagle Medium) containing 1% L‐Glutamine, 1% penicillin/streptomycin, and 10% fetal calf serum (FCS). Empty wells without any sample were used as negative controls. The extraction process was carried out at 37 °C with 100% humidity and 5% CO_2_ for 24 h. NHDFs were seeded with 10 000 cells per well (TPP Techno Plastic Products AG, Trasadingen, Switzerland) in 100 µL DMEM supplemented with 1% L‐Glutamine, 1% penicillin/streptomycin and 10% fetal calf serum 1 day before incubation with extracts. The NHDFs were then incubated for 24 h with 100 µL extracts by replacing the old media. The viable NHDFs cells of negative control were set as 100%, and the ones incubated with 1% Triton X‐100 in supplemented DMEM were regarded as the positive control. Cell viability of the NHDFs was determined via MTS assay by measuring the absorbance at 490 nm.

### Skin Irritation and Sensitization

A KeratinoSens skin sensitization & irritation assay was performed according to OECD guideline No. 442D,^[^ ^63^ ^]^ described in detail described in ref. [64] in order to assess the acute in vitro skin toxicity of the catalytic plasma coatings (np‐SiOx/AgOx/TiOx; AgOx/TiOx) substrates. To mimic a more realistic exposure scenario, artificial sweat^[^ ^64^ ^]^ was applied as an extract solvent during an extraction conducted at 37 °C for 24 h (± 1 h) on an orbital shaker set to 300 rpm in darkness. Extraction volume ratio is determined according to ISO10993‐12.^[^ ^65^ ^]^ In short, positive control: EGDMA (1–1′000 µm) and artificial sweat catalytic plasma coatings sample extracts (0.005–10%) were contacted for 48 h onto KeratinoSens skin cells maintained in DMEM assay media in 96 well plates. Skin irritation (cell viability) was assessed with AlamarBlue (Invitrogen, DAL1100, excitation 540 nm / emission 590 nm) whereas skin sensitization (fold‐induction) was evaluated with One‐Glo Reagent (Promega, E6120, integration time = 1 s/well). Subsequently, EC_1.5_ values (fold‐induction) and IC_50_ values of dose‐dependent controls and samples were calculated according to OECD guideline No. 442D.^[^ ^63^ ^]^

### Antibacterial Activity

The antibacterial activity of the catalytic plasma coatings was qualitatively assessed by touch test and quantitatively by a modified ASTM method. The samples were deposited on glass (10 mm). *Escherichia coli* (E. coli) DSM 1003 and *Staphylococcus aureus* (S. aureus) ATCC 6538 were selected as gram‐positive and gram‐negative representative pathogens, respectively. Three replicates were analyzed for each measurement. For the qualitative (touch test) antibacterial assay, bacteria colonies from the respective agar plate stock were cultured overnight in 5 mL Tryptic Soy Broth (TSB) supplemented with 0.25% Glucose media at 37 °C with agitation (160 rpm). The overnight culture was diluted with TSB to 0.1 of optical density at 600 nm (OD600) and regrown for 1.5–2 h to reach exponential growth. Thereafter, the regrown cultures were diluted in TSB to a final OD600 nm of 0.05 for *S. aureus* and of 0.1 for *E. coli*. 100 µL of the resulting suspension was plated on Plate Count Agar (PC‐agar) and let dry for 30 min in the air. The catalytic plasma coating samples were placed onto these agar plates, and allowed interaction for 10 and 60 min. The agar plates were then incubated at 37 °C for overnight, and the resultant growth inhibition was visualized and documented photographically using SCAN300 (Interscience, France).

The antibacterial efficacy was further measured quantitatively for both bacterial strains following a modified ASTM method.^[^ ^27^, ^49^ ^]^ Bacteria colonies from the respective agar plate stock were again cultured overnight in 5 mL TSB and glucose media as described above. After overnight culture and reaching exponential growth, regrown cultures were diluted to a final OD600nm of 0.01 in 0.9% NaCl. Therefrom, 100 µL was loaded onto the samples as well as the controls. The samples were then incubated for 60 min at RT without shaking. The suspension was subsequently removed, and the samples were washed twice with 1 mL 0.9% NaCl to remove the adhered bacteria. Then, removed bacterial suspension and the washing solution from each sample was collected and mixed. The washed samples were placed in 2.5 mL 1 x PBS, sonicated for 5 min, and thereafter vigorously vortexed for 15 s. Serial dilutions of the two collected bacterial mixtures were spotted on PC agar plates, then incubated at 37 °C overnight. After incubation, bacterial colonies were counted to obtain an estimation of the viable cells on each of the samples.

### Antiviral InVIS Membrane Integrity

The inactivated virus membrane integrity assay was performed according to the literature^[^ ^54^ ^]^ with minor modifications. AgOx/TiOx and np‐SiOx/AgOx/TiOx samples deposited on glass (1 cm diameter) were tested, as well as bare glass substrates and the addition of positive control (OEG, 1.25 mg mL^−1^). In short, 20 µL InVIS solution was dropcasted onto the samples and incubated for 20 min. Then, each sample was transferred into falcon tubes containing 20 mL of PBS and underwent agitation to ensure the homogenization of the freshly added PBS and the remaining inoculum. After mixing, 2 mL of the washout was collected and pipetted into transparent cuvettes. The emission fluorescence spectra of the washout were characterized using the Horiba FluoroMax SpectraFluorometer. Excitation wavelength was set to 560 nm and the emission intensity was measured between 580 and 650 nm.

### Antiviral MHV Activity

The antiviral activity was evaluated according to ISO 18184:2019(E),^[^ ^66^ ^]^ described in more detail in Ref. [64]. This assessment focused on the inactivation potential of the catalytic plasma coatings (AgOx/TiOx and np‐SiOx/AgOx/TiOx) against the MHV (Coronavirus, Group 2, ATCC VR1426) over an exposure period of 2 h. Plaque assays applying L929 murine subcutaneous connective tissue host cells were conducted in independent triplicates (N = 3, *n* = 2). Negative controls and immediate washout were performed with non‐plasma treated glass surface carriers. Subsequently, in accordance with ISO 18184 guideline,^[^ ^66^ ^]^ the antiviral activity was determined.

### Statistical Analysis

The data were processed using the relevant instruments and subsequently normalized and analyzed with Origin 2022. EPR measurements were specifically evaluated using MATLAB. Results are presented as mean values, with standard deviations included where applicable. All experiments were repeated at least three times using three replicate samples per experiment. Depending on the analytical technique, statistical analysis was performed using Excel, Origin 2022, or MATLAB.

## Conflict of Interest

The authors declare no conflict of interest.

## Supporting information



Supporting Information

## Data Availability

The data that support the findings of this study are available from the corresponding author upon reasonable request.
