# Supplementary material for: Plasma Functionalization Enables Diffusion Control of Reactive Oxygen Species
Source: Small. 2025 Jul 11;21(35):2502311. doi: 10.1002/smll.202502311 (PMC12410902; doi:10.1002/smll.202502311)
Supplement: Supplementary file 1 — Supporting Information [file SMLL-21-2502311-s001.docx]

**Supporting Information**

**Plasma Functionalization Enables Diffusion Control of Reactive Oxygen Species**

Paula Navascués ^a,^*, Flaela Kalemi ^a^, Flavia Zuber ^b^, Philipp Meier ^c^, Ludovica M. Epasto ^d^, Michal Góra ^a,e^, Barbara Hanselmann ^a^, Svetlana Kucher ^d^, Enrica Bordignon ^d^, Qun Ren ^b^, Giacomo Reina ^c,^*, Dirk Hegemann^a,^*.

*^a^ Laboratory for Advanced Fibers, Empa, Swiss Federal Laboratories for Materials Science and Technology, Lerchenfeldstrasse 5, 9014, St. Gallen, Switzerland.*

*^b^ Laboratory for Biointerfaces, Empa, Swiss Federal Laboratories for Materials Science and Technology, Lerchenfeldstrasse 5, 9014, St. Gallen, Switzerland.*

*^c^ Particles-Biology Interactions Laboratory, Empa, Swiss Federal Laboratories for Materials Science and Technology, Lerchenfeldstrasse 5, 9014, St. Gallen, Switzerland.*

*^d^ Department of Physical Chemistry, University of Geneva, 30 Quai Ernest Ansermet, 1211 Genève 4, Switzerland.*

*^e^ Department of Materials, ETH Zürich, 8093 Zürich, Switzerland.*

Corresponding authors (*): [paula.denavascues@empa.ch](mailto:paula.denavascues@empa.ch) (P. Navascués), [giacomo.reina@empa.ch](mailto:giacomo.reina@empa.ch) (G. Reina), [dirk.hegemann@empa.ch](mailto:dirk.hegemann@empa.ch) (D. Hegemann).

This supporting information includes 6 figures and 1 table.

**S1**. **FIB-cross section SEM analysis of np-SiOx/AgOx/TiOx**

**Figure** **S1** shows a FIB-SEM analysis of a np-SiOx/AgOx/TiOx with a tilt angle of 52° and detecting backscattered electrons at 3 kV. Backscattered electrons allow to observe the contrast between the Si wafer (marked with blue shallow), the AgOx/TiOx layer (violet) and the npSiOx functional layer (red). An additional C-coating (yellow) was deposited on top of the sample to be able to perform the cross section with FIB, allowing to subsequent processing without damaging the surface of the sample. AgOx islets can be distinguished because of their brighter contrast. Approximate thickness of the different layers is 55±5 nm for AgOx/TiOx and 40 nm for the np-SiOx layer on top. The FIB-SEM measurements were conducted using TFS Helios 600i device.


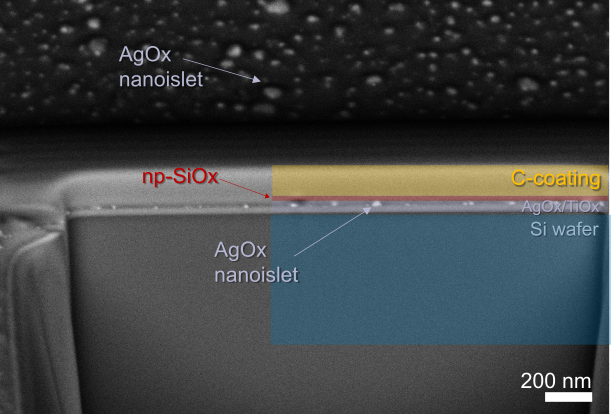


**Figure S1**. SEM-FIB characterization of np-SiOx/AgOx/TiOx (BSE detected at 3 kV, tilt angle 52°).

**S2. Water contact angle measurements of AgOx/TiOx and np-SiOx/AgOx/TiOx samples.**

Water contact angle (2 µL, mQ water) was measured in static mode for samples deposited on glass, showing a hydrophilic wetting of the catalytic plasma coating (i.e., AgOx/TiOx), with a contact angle of around 60°, see **Figure S2 (a)**. On the other hand, the sample functionalized with the nanoporous SiOx layer (i.e., np-SiOx/AgOx/TiOx) shows a superhydrophilic behaviour, with a WCA below 10° and the water droplet quickly seeping, see **Figure S2 (b)**.


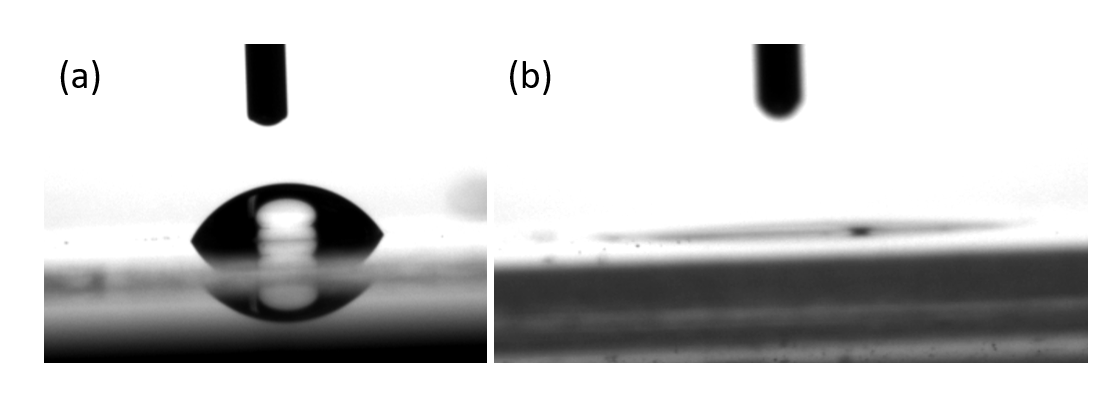


**Figure S2**. Water contact angle of (a) AgOx/TiOx and (b) np-SiOx/AgOx/TiOx coatings deposited on glass.

**S3. Total and diffuse transmittance and diffuse reflectance of AgOx/TiOx and np-SiOx//AgOx/TiOx plasma coatings.**

**Figure S3** shows UV-Vis characterization that complement the data shown in Figure 2 in the manuscript. The antireflective property of the thin np-SiOx layer (7 nm) does not alter the total transmittance of the system, as shown in **Figure S3 (a)**, with similar total transmittance for both plasma coatings. Indeed, as shown in Figure 2 of the main text, the reduction in reflectance leads to an increase in absorptance. On the other hand, the films exhibit good optical quality, as demonstrated by the low values of diffuse components for diffuse transmittance and reflectance, as observed in **Figure S3 (b)** and **(c)**, respectively.


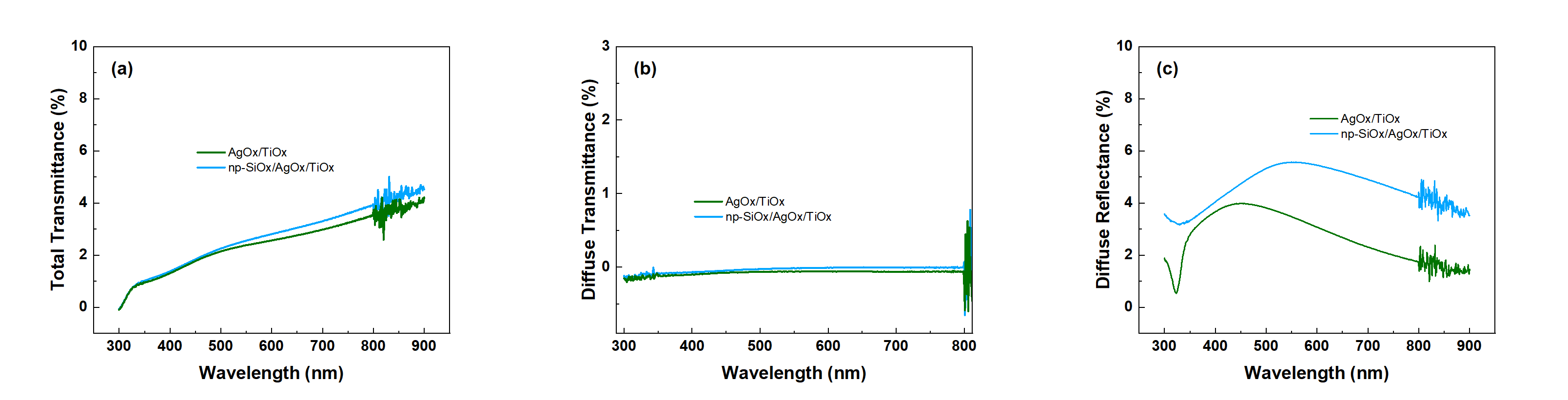


**Figure S3.** (a) Total transmittance, (b) diffuse transmittance, and (c) diffuse reflectance of AgOx/TiOx and np-SiOx/AgOx/TiOx plasma coatings (np-SiOx thickness: 7 nm; AgOx/TiOx thickness: 55 ± 5 nm).

**S4. Pseudo-first order degradation kinetic analysis of DHE by catalytic plasma coatings.**


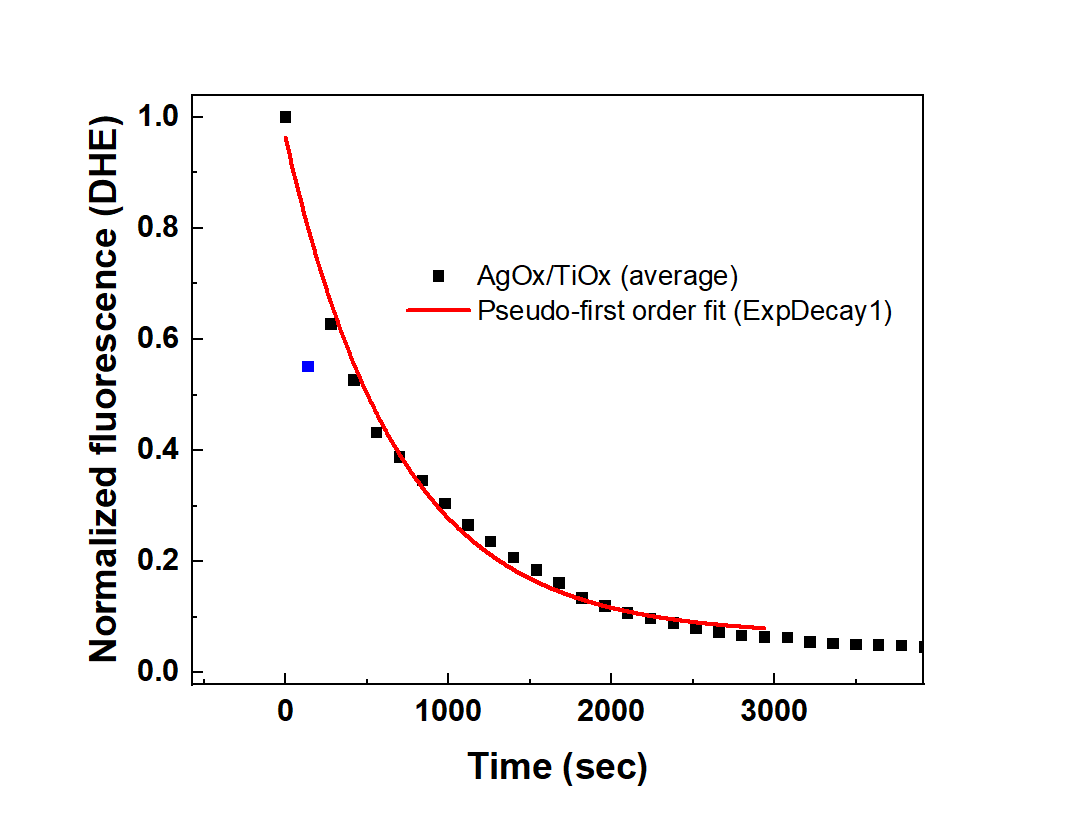
The degradation kinetics of dihydroethidium (DHE) can be fitted as a pseudo first-order kinetic, as shown in **Figure** **S4** (experimental points plotted in black and fitting as a red line). The rate constants *(k*) discussed in the main text have been calculated as the inverse of the fitted parameter *t*. Analysis of the different samples has been conducted similarly, fixing *y0*=0, *x0*=0 and A_1_=1, thereby determining the different *t* constants. Deviated experimental point, mainly caused by bubbles on top of the sample, such as this plotted in blue in **Figure S4**, are discarded for the fitting analysis. **Table S1** include relevant information of the analysis.

| **Model** | ExpDecay1 |
| --- | --- |
| **Equation** | y=exp(-x/t) |
| ***t* [s]** | 818 ± 27 |
| ***k*=1/t [s-1]** | ~1.4·10^-3^ |
| **R-square** | 0.9708 |

**Figure S4 and Table S1**. Analysis of DHE degradation kinetic, showing experimental points fitted data, as well as the table with the relevant fitting parameters.

**S5. DHE degradation experiment in anoxic conditions.**

To confirm that superoxide is formed by O_2_ reduction, DHE degradation experiments were comparatively performed under anoxic and oxic conditions, as shown by the blue points and red line, respectively, in **Figure S5**. Deoxygenated conditions in DHE solution were achieved after at least three freeze-thaw cycles using a Schlenk line. The results confirm that clear degradation of DHE requires O_2_ in solution. Note that this experiment was performed using a different device than the one used to acquire the data presented in the manuscript (in that case, a SynergyH1 plate reader). In this case, experiments were conducted using a Horiba FluoroMax-Plus spectrofluorometer, with the sample placed at the bottom of 4.5 mL cuvettes and 3 mL of a 10 µm DHE solution pipetted in.


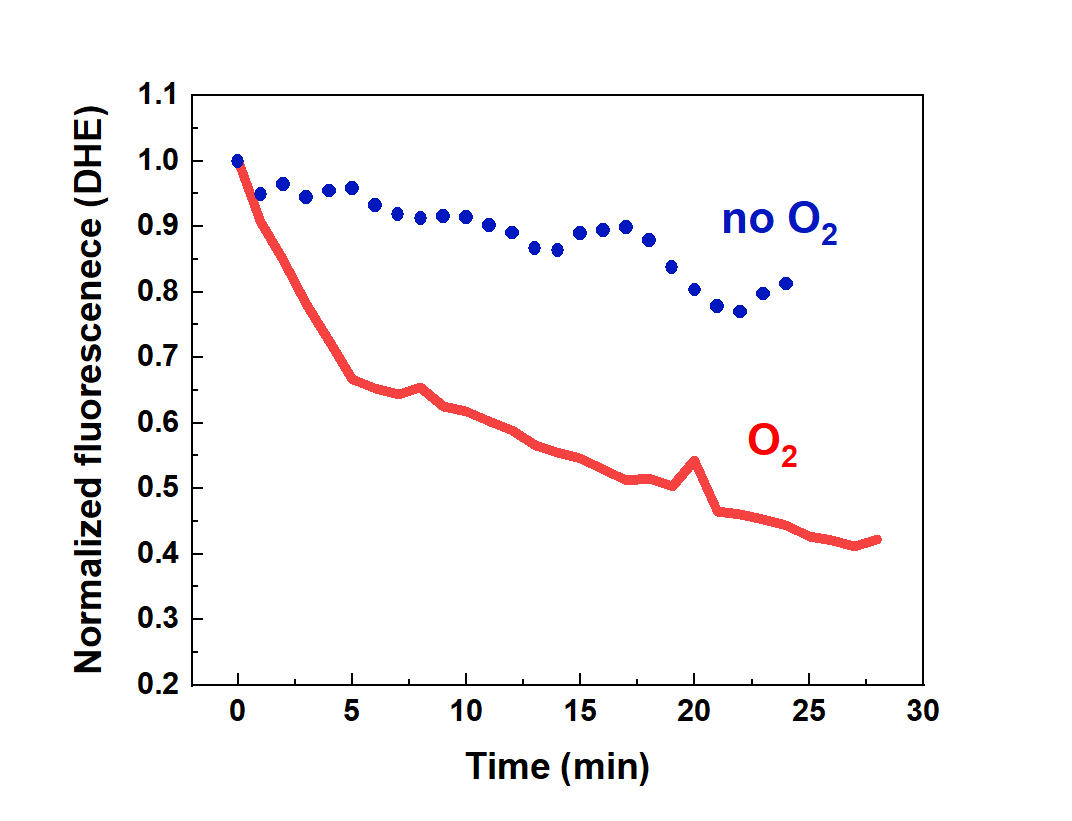


**Figure S5.** DHE degradation in anoxic (no O_2_, blue points) and oxic (O_2_, red line) conditions for the AgOx/TiOx catalytic plasma coating.

**S6. Additional EPR information**


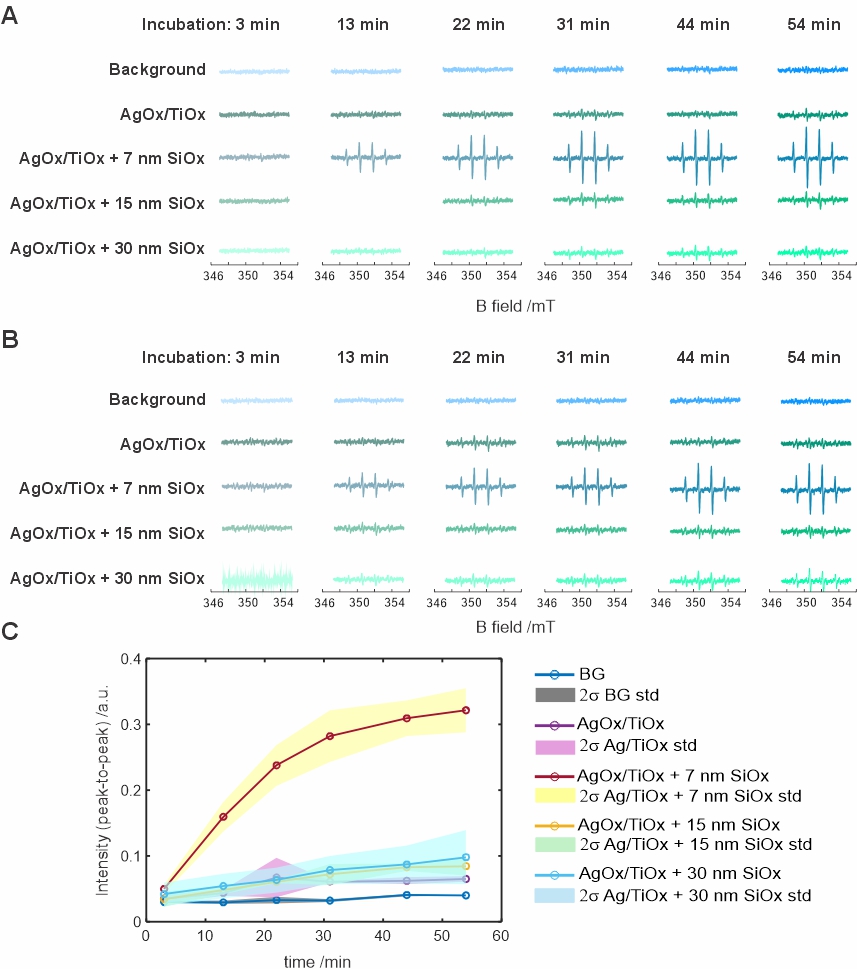


**Figure S6.** EPR experiments at different incubation times for the catalytic plasma coatings. First (A) and second (B) repetition of the spin trapping experiments with 100 mM of DMPO on different samples indicated in the figure. C) Evolution of the averaged intensity peak-to-peak of the two central peaks in the EPR spectrum versus time. The highest increase in intensity is for the 7 nm np-SiOx/AgOx/TiOx.
